# Astragalus polysaccharides augment BMSC homing via SDF-1/CXCR4 modulation: a novel approach to counteract peritoneal mesenchymal transformation and fibrosis

**DOI:** 10.1186/s12906-024-04483-5

**Published:** 2024-05-24

**Authors:** Funing Wang, Huibo Dai, Ziren Zhou, Yun Shan, Manshu Yu, Jinyi Sun, Li Sheng, Liyan Huang, Xiaohui Meng, Yongqing You, Meixiao Sheng

**Affiliations:** 1 Department of Nephrology, Affiliated Hospital of Nanjing University of Chinese Medicine (Jiangsu Province Hospital of Chinese Medicine), Nanjing, China; 2 Medical Experimental Research Center, First College of Clinical Medicine, Nanjing University of Chinese Medicine, Nanjing, China

**Keywords:** Astragalus polysaccharide, Bone marrow mesenchymal stromal cells, Peritoneal fibrosis, SDF-1/CXCR4 axis, Cell homing

## Abstract

**Purpose:**

This study aimed to evaluate the potential of astragalus polysaccharide (APS) pretreatment in enhancing the homing and anti-peritoneal fibrosis capabilities of bone marrow mesenchymal stromal cells (BMSCs) and to elucidate the underlying mechanisms.

**Methods:**

Forty male Sprague-Dawley rats were allocated into four groups: control, peritoneal dialysis fluid (PDF), PDF + BMSCs, and PDF + ^APS^BMSCs (APS-pre-treated BMSCs). A peritoneal fibrosis model was induced using PDF. Dil-labeled BMSCs were administered intravenously. Post-transplantation, BMSC homing to the peritoneum and pathological alterations were assessed. Stromal cell-derived factor-1 (SDF-1) levels were quantified via enzyme-linked immunosorbent assay (ELISA), while CXCR4 expression in BMSCs was determined using PCR and immunofluorescence. Additionally, a co-culture system involving BMSCs and peritoneal mesothelial cells (PMCs) was established using a Transwell setup to examine the in vitro effects of APS on BMSC migration and therapeutic efficacy, with the CXCR4 inhibitor AMD3100 deployed to dissect the role of the SDF-1/CXCR4 axis and its downstream impacts.

**Results:**

*In vivo and in vitro* experiments confirmed that APS pre-treatment notably facilitated the targeted homing of BMSCs to the peritoneal tissue of PDF-treated rats, thereby amplifying their therapeutic impact. PDF exposure markedly increased SDF-1 levels in peritoneal and serum samples, which encouraged the migration of CXCR4-positive BMSCs. Inhibition of the SDF-1/CXCR4 axis through AMD3100 application diminished BMSC migration, consequently attenuating their therapeutic response to peritoneal mesenchyme-to-mesothelial transition (MMT). Furthermore, APS upregulated CXCR4 expression in BMSCs, intensified the activation of the SDF-1/CXCR4 axis’s downstream pathways, and partially reversed the AMD3100-induced effects.

**Conclusion:**

APS augments the SDF-1/CXCR4 axis’s downstream pathway activation by increasing CXCR4 expression in BMSCs. This action bolsters the targeted homing of BMSCs to the peritoneal tissue and amplifies their suppressive influence on MMT, thereby improving peritoneal fibrosis.

**Supplementary Information:**

The online version contains supplementary material available at 10.1186/s12906-024-04483-5.

## Introduction

Peritoneal dialysis (PD) serves as a crucial renal replacement therapy for patients with end-stage renal disease, providing benefits such as the preservation of residual kidney function, ease of use, cost-effectiveness, significant societal benefits, and survival rates comparable to those of hemodialysis [1]. However, the sustainability of PD is compromised by the progressive deterioration of the peritoneal membrane’s structure and function. Peritoneal mesothelial cells (PMCs), the initial barrier encountering peritoneal dialysis fluid (PDF), are among the first to incur damage. They initiate the secretion of inflammatory cytokines and undergo a mesothelial-mesenchymal transition (MMT), leading to the eventual development of peritoneal fibrosis (PF) [2, 3]. Hence, formulating new approaches to modulate PMC-MMT and mitigate PF is essential for the prolonged viability of peritoneal dialysis.

Mesenchymal stem cells (MSCs), particularly those derived from bone marrow (BMSCs), have garnered significant attention as potential therapeutic agents for a variety of fibrotic diseases. Their appeal lies in their capacity for multidirectional differentiation, robust proliferative abilities, minimal immunogenicity, potent tissue repair capabilities, and intrinsic chemotactic homing properties [4, 5]. BMSCs have been documented to navigate to sites of injury and facilitate the structural and functional restitution of tissues and organs via paracrine actions in conditions such as post-infarction fibrosis, cirrhosis, and renal fibrosis [6, 7]. Nevertheless, the therapeutic potential of BMSCs is often hampered by suboptimal engraftment rates. Emerging research indicates that pre-treatment strategies could substantially bolster the therapeutic efficacy of MSCs [8].

The SDF-1/CXCR4 axis is pivotal in the homing mechanism of stem cells and has become increasingly relevant in addressing fibrotic diseases [9–11]. Interaction between SDF-1 and CXCR4 triggers a cascade of intracellular signaling pathways, notably MAPK and PI3K/Akt, which influence cell motility and migration [12–14]. This activation is critical for the targeted homing of BMSCs to fibrotic sites and their subsequent anti-fibrotic effects.

Recent research on medicinal plants indicates that phytochemicals can boost the expression of the SDF-1/CXCR4 axis [15]. Astragalus polysaccharide (APS), a principal constituent of the traditional Chinese herb Huangqi, has been demonstrated to mitigate inflammation and fibrosis by blocking NF-κB and inhibiting the TGF-β1/SMADs pathway [16–18]. Moreover, a series of studies have revealed that the major active ingredient of Astragalus membranaceus, when applied to MSCs, enhances cell proliferation, restores impaired functions, and increases cell homing to ischemic cardiac sites [19–21]. These findings suggest that APS intervention may augment the quantity and survival rate of bone marrow mesenchymal stem cells during their homing process and ameliorate the repair of damaged tissues.

This study aimed to evaluate whether APS pre-treatment enhances the homing capacity and anti-fibrotic efficacy of BMSCs in the peritoneum and to elucidate the underlying mechanisms. Utilizing both in vitro and in vivo approaches, we examined the influence of APS on the migration and homing behaviors of BMSCs, with a particular emphasis on the modulation of the SDF-1/CXCR4 axis by APS. The outcomes of this research hold substantial scientific and clinical relevance, offering deeper insights into the mechanistic role of APS in stem cell therapy and advancing the therapeutic application of BMSCs for the management of PF.

## Materials and methods

### Isolation, culture, and characterization of BMSCs

Male Sprague-Dawley (SD) rats (aged 4–5 weeks old, weighing 100–120 g) were acquired from Beijing Vital River Experimental Animal Technology Co., Ltd. BMSCs were harvested from the femurs and tibias of these rats by flushing with DMEM/F12 medium (Gibco, CA, USA). These cells were then cultured in an environment with 5% CO_2_ at 37 °C. After 48 h, non-adherent cells were removed, and the medium was refreshed every two to three days. Upon reaching approximately 80% confluence, cells were detached using trypsin and subcultured. The third passage of BMSCs was characterized by flow cytometry using antibodies against CD29 (Thermo-Fisher Scientific, Bremen, Germany), CD90, CD45, and CD11b (Biolegend, CA, USA). The differentiation of BMSCs into adipocytes and osteocytes was induced using Oricell commercial kits (Guangzhou, China), verifying their stem cell properties. BMSCs from passages 2 to 5 were utilized for the experimental studies.

### Isolation, culture, and characterization of PMCs

Under sterile conditions, peritoneal tissue was harvested from the retinas of SD rats and finely minced. The tissue was then digested using 0.25% trypsin (Gibco) at 37 °C for 25 min. The digestion was halted by adding DMEM/F12 medium (Gibco) supplemented with 10% fetal bovine serum (FBS). Subsequently, the cell suspension was filtered through a 70 μm cell strainer, and cells were collected by centrifugation at 1000 rpm for 5 min. The resultant cell pellet was resuspended in DMEM/F12 medium (Gibco) enhanced with 10% FBS, 100 IU/ml penicillin, and 100 µg/ml streptomycin, to a total volume of 4 ml. This suspension was then plated in a 25 cm² tissue culture flask (NEST Biotechnology Co., Ltd., Wuxi, China). After 24 h of incubation at 37 °C, the medium was replaced, and cellular confluence was typically observed within 5–7 days. To identify primary PMCs, immunocytochemical staining was performed using antibodies against vimentin (1:1000, CST, MA, USA) and pan-Cytokeratin (1:1000–4000, Proteintech, Wuhan, China).

### Transmembrane migration experiment

An in vitro cell migration assay was established using a transmembrane chamber system equipped with a membrane filter featuring 8-micrometer pores, provided by Labselect (Beijing, China). Before initiating the experiment, BMSCs underwent a 24-hour pre-treatment with or without APS at a concentration of 1 mg/mL. These cells were then placed in the upper chamber of a 24-well plate (NEST Biotechnology Co., Ltd., China) at a density of 2 × 10^4^ cells per well in a culture medium. The lower chamber was supplemented with varying concentrations of SDF-1 (0, 25, 50, 100, or 150 ng/mL). For inhibition treatment, BMSCs were pre-treated with AMD3100 (Plerixafor, a CXCR4-specific inhibitor) (MCExpress, New Jersey, USA) for 1 h prior to the migration assay. Cells migrating to the lower side of the filter were stained with crystal violet, and five random fields (200× magnification) were chosen for cell counting under an Olympus microscope (Tokyo, Japan).

In a 6-well plate (NEST Biotechnology Co., Ltd.), the lower chamber was filled with 2 × 10^5^ PMCs, treated with or without 3% PDF (Deerfield, IL, USA). The upper chamber was seeded with BMSCs at a comparable density. For inhibitor treatment, BMSCs were pre-treated with AMD3100 (25 µg/mL) for 1 h prior to transplantation. Following a 24-hour migration period, the transwell apparatus was washed, and non-migrated BMSCs were removed from the upper side of the membrane using a cotton swab. Migrated BMSCs were subsequently stained and evaluated under a microscope, with five random fields of view analyzed at 40× magnification.

### Animals

Forty male SD rats were randomly allocated into four groups: control, PF, PF + BMSCs, and PF + ^APS^BMSCs, each containing ten rats. To induce the PF model, the PF group received daily intraperitoneal injections of 10 mL/100 g PDF over four weeks. In contrast, the control group was administered an equivalent volume of normal saline. Following this, rats in the PF + BMSCs and PF + ^APS^BMSCs groups were given weekly tail vein injections of 0.3 mL cell suspension containing 3 × 10^6^ BMSCs or ^APS^BMSCs, respectively. Concurrently, rats in the control and PF groups received 0.3 mL of phosphate-buffered saline (PBS) instead. All rats were euthanized 24 h post the final cell transplantation under sodium pentobarbital anesthesia for sample collection. This animal study received approval from the Animal Ethics Committee of Nanjing University of Chinese Medicine (ACU230205) and was conducted following the Guide for the Care and Use of Laboratory Animals (National Institutes of Health, USA, 1985).

### Tracking study of Dil-labeled BMSCs after transplantation

Twelve SD rats were randomly assigned into control, PDF + BMSCs, and PDF + ^APS^BMSCs, with each group comprising four rats. The PF model was induced through daily intraperitoneal injections of 10 mL/100 g PDF for four weeks. Prior to transplantation, BMSCs and ^APS^BMSCs were labeled with DiI dye (Beyotime Biotechnology, Shanghai, China). These fluorescently labeled cells were subsequently administered to the rats via tail vein injection. To assess the homing of BMSCs to the peritoneal tissue, peritoneal tissues were harvested 24 h post-transplantation and processed into frozen sections. These sections were fixed, washed, and then incubated with DAPI (Beyotime Biotechnology) for nuclear staining. Finally, the sections were visualized and photographed using a fluorescence microscope (Nikon Corporation, Tokyo, Japan).

### Histology and immunohistochemical (IHC) analysis of peritoneal samples

Histological and IHC analyses were performed on samples obtained from the lateral peritoneal wall to evaluate the pathological condition and thickness of the peritoneum. Initially, the samples were fixed in 4% formaldehyde for 24 h, followed by dehydration and paraffin-embedding. The embedded samples were then sectioned into 5-micrometer slices for analysis. To assess the pathological condition and thickness, we employed hematoxylin and eosin (H&E) staining, as well as Masson’s trichrome staining, for detailed histological examination. For IHC analysis, the sections were incubated with primary antibodies targeting collagen (1:1200, Abcam, Cambridge, UK) and fibronectin (1:800, CST, Massachusetts, USA) and then with corresponding secondary antibodies.

### Immunocytochemical staining of PMCs and BMSCs

Immunocytochemical staining was employed to identify PMCs and to verify CXCR4 expression in BMSCs through immunofluorescence staining. Initially, cells were fixed, washed, and permeabilized using Triton X-100 (Beyotime Biotechnology) for 30 min. Following this, cells underwent a blocking phase for 1 h with 5% bovine serum albumin (BSA) (BioFroxx, Einhausen, Germany) to prevent nonspecific binding. Cells were then incubated overnight at 4 °C with the following primary antibodies: vimentin (1:100, CST), cytokeratin (1:100, Proteintech, Wuhan, China), and CXCR4 (1:100, Proteintech). Post-primary antibody incubation, cells were washed and subsequently incubated with fluorescently labeled secondary antibodies, Alexa Fluor 594 goat anti-rabbit IgG (1:100, Proteintech) and Alexa Fluor 488 goat anti-mouse IgG (1:100, Proteintech), for 2 h to enable visualization. Finally, nuclear staining was conducted using DAPI, and the cells were examined under fluorescence inverted microscopy.

### Enzyme-linked immunosorbent assay (ELISA) for detecting SDF-1 levels

Samples were collected from both the control and PDF groups to assess the levels of SDF-1 in the peritoneal and serum samples of rats. Peritoneal tissues were homogenized, and the resultant supernatant was harvested for analysis. The SDF-1 concentration in these samples was quantified using an ELISA assay kit (AiFang Biotechnology, Wuhan, China). In short, samples and standards were prepared, followed by their addition to the ELISA plate for incubation. After the incubation period, the plate was washed to remove unbound substances. A substrate solution was then added to initiate a colorimetric reaction, which was subsequently halted by adding a stop solution. The absorbance was measured to determine the SDF-1 concentration in the samples.

### Analysis of protein expression changes in co-cultured PMCs and BMSCs using the western blot method

To examine the alterations in protein expression resulting from co-culture with BMSCs, PMCs were initially plated in a 6-well plate at a density of 2 × 10^5^ cells per well. Following overnight adherence, the cells were exposed to 3% PDF for 24 h and subsequently co-cultured with BMSCs. Post 24-hour co-culture, PMCs from the lower chamber were lysed using ice-cold RIPA buffer (Thermo Fisher Scientific, Massachusetts, USA) supplemented with 2% protease and phosphatase inhibitors (Beyotime Biotechnology). The lysates were then subjected to sonication and centrifugation, and the supernatants were collected. Protein concentrations were determined utilizing a protein assay kit (Beyotime Biotechnology). The proteins (20 µg per sample) were then separated by SDS-PAGE and electroblotted onto PVDF membranes. These membranes were blocked using PBS containing 5% BSA, followed by overnight incubation at 4 °C with specific primary antibodies. Subsequent incubation with horseradish peroxidase-conjugated secondary antibodies (mouse/rabbit IgG) was performed for one hour. Protein bands were detected using the enhanced chemiluminescence system (Biosharp, Anhui, China) and visualized with a chemidoc imaging system. The primary antibodies employed in this study included E-cadherin (1:1000–4000, Proteintech), α-smooth muscle actin (α-SMA) (1:1000–8000, Proteintech), vimentin, and β-actin (1:1000, Santa Cruz Biotechnology, USA), and secondary antibodies were mouse/rabbit IgG conjugated with horseradish peroxidase (1:20000, Zhongshanjinqiao, Beijing, China).

### Analysis of gene expression in peritoneum and PMCs using real-time quantitative PCR technique

To evaluate gene expression in the peritoneum and PMCs, total RNA was isolated from these samples employing TRIzol reagent (Vazyme Biotech Co., Ltd., Nanjing, China). The extracted RNA was then reversely transcribed into cDNA using the All-In-Two RT SuperMix for qPCR (Vazyme Biotech Co., Ltd., Nanjing, China). Quantitative real-time PCR (qPCR) analyses were conducted using the ABI PRISM 7500 system (Applied Biosystems, New York, USA) with SYBR Green as the detection dye. The specific gene sequences assessed are detailed in Table 1.

**Table 1:** List of Primers Used for qRT-PCR in This Study

Gene	Forward sequence (5’-3’)	Reverse sequence (5’-3’)
β-actin	TCACCCACACTGTGCCCATCTATGA	CATCGGAACCGCTCATTGCCGATAG
vimentin	AATGCTTCTCTGGCACGTCT	GTGAGGTCAGGCTTGGAAAC
α-SMA	ATCCGATAGAACACGGCATC	CATACATGGCAGGGACATTG
E-cadherin	CCTACAATGCTGCCATCGCCTAC	GGGTAACTCTCTCGGTCCAGTCC
CXCR4	CATACATGGCAGGGACATTG	CCTACAATGCTGCCATCGCCTAC
SDF-1	GGGTAACTCTCTCGGTCCAGTCC	TCACCCACACTGTGCCCATCTATGA

### Statistical analysis

In this study, data analysis and graphical representations were performed using GraphPad Prism software (version 9.5, GraphPad Software Inc., California, USA) and SPSS software (version 26.0, IBM Corp., Armonk, USA). All data are presented as the mean ± standard deviation. Statistical analyses were conducted using students’ t-tests or one-way analysis of variance (ANOVA) for comparisons of measured data. Post-hoc comparisons among multiple groups were facilitated using the Least Significant Difference (LSD) test. *P* < 0.05 was considered to indicate statistical significance.

## Results

### Identification and differentiation potential of BMSCs: in vitro culture and surface marker analysis

**Fig. 1:**
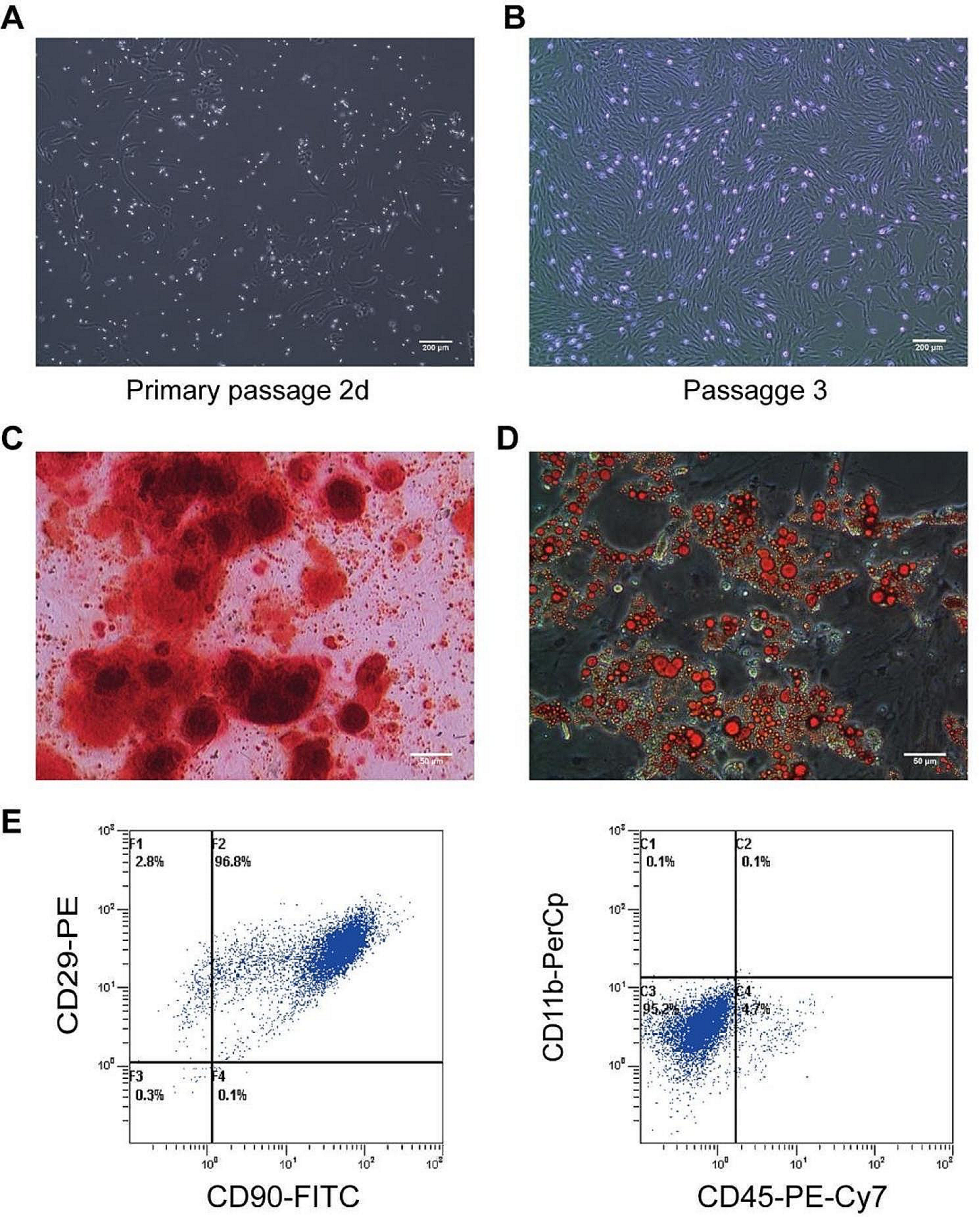
Identification of BMSCs. (**A-B**) Morphological characteristics of BMSCs (×40). (**C**) After staining with Alizarin Red S, abundant calcium deposits were visualized in the osteoblasts. (**D**) After Oil Red O staining, lipid droplets were observed in the cytoplasm of adipocytes (×200). (**E**) Flow cytometry was used to analyze and identify surface markers of BMSCs, including CD90, CD29, CD45, and CD11b

After 48 h of culture, BMSCs demonstrated a typical spindle-shaped morphology, forming tight adhesions and individual colonies. By approximately 7 to 9 days, the BMSCs achieved 80–90% confluence, displaying a helical arrangement in alignment with the criteria established by the International Society for Cellular Therapy (ISCT) [22]. The passaged cells retained a fibroblast-like morphology (Fig. 1A-B). Flow cytometric analysis revealed that in the third passage, BMSCs expressed surface markers CD90 and CD29 at rates of 94.63%±6.23% and 98.82%±1.16%, respectively. In contrast, the expression rates for CD45 and CD11b were notably lower, at 5.34%±0.64% and 0.2%±0.07%, respectively, confirming the characteristic phenotypic features of BMSCs (Fig. 1E). Moreover, in vitro induction assays revealed that BMSCs could differentiate into adipocytes and osteocytes, as evidenced by Oil Red O and Alizarin Red S staining, respectively (Fig. 1C-D). These findings demonstrate that BMSCs cultured under specific conditions not only exhibit morphological characteristics following ISCT standards but also retain the capacity to differentiate into adipocytes and osteocytes.

### APS enhances the ability of BMSCs to improve PF in a rat model

Following the validation of the morphological characteristics and differentiation capabilities of BMSCs, our study proceeded to explore the therapeutic potential of ^APS^BMSCs in a rat PF model. PF typically occurs when the peritoneum undergoes significant damage, leading to enhanced proliferation and repair processes in the interstitial fibrous connective tissue, which culminates in fibrotic lesions. Consequently, the severity of PF can serve as a direct indicator of peritoneal injury as well as the efficacy of stem cell-based treatments. Our experimental findings indicated that BMSCs treated with 1 mg/mL APS displayed the highest level of cellular vitality (Figure S1). Based on these results, this concentration was chosen for subsequent experiments to assess the therapeutic effects of ^APS^BMSCs on PF.

**Fig. 2:**
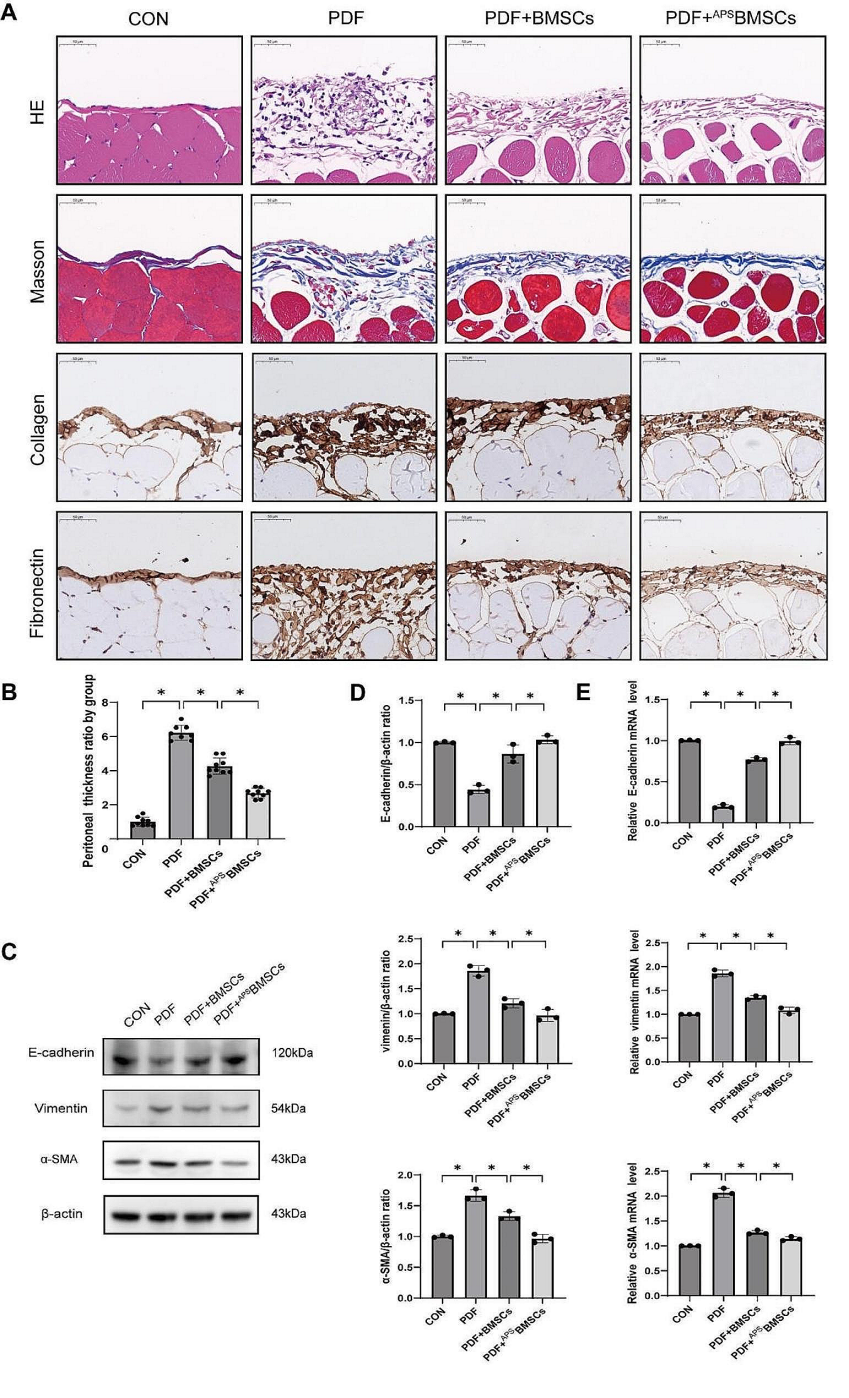
APS enhances the ability of BMSCs to improve peritoneal fibrosis in rats. (**A**) HE staining of the rat peritoneum revealed the accumulation of inflammatory cells. Peritoneal thickness was quantitatively assessed using Masson’s trichrome staining. Immunohistochemical staining showed the expression of collagen and fibronectin in the peritoneum (scale bar = 50 μm). (**B**) The quality statistical results of Masson’s trichrome staining were represented by a histogram. (**C**) Western blot analysis was performed to evaluate the expression of MMT markers in peritoneal tissue treated with 4.25% PDF, with or without injection of BMSCs or ^APS^BMSCs. (**D**) The quality statistical results of Figure C are represented by a histogram. (**E**) qRT-PCR analysis of E-cadherin, vimentin, and α-SMA mRNA expression levels in PMCs. The markers are not capable of displaying the images, so the original images for the full-length prints are not available; the original images for all prints (with the edges of the film clearly visible) have been provided in the Supplementary Information document

In comparison to the control group, significant differences were noted in the peritoneum subjected to PDF damage, as evidenced by HE and Masson’s trichrome staining (Fig. 2A). The PDF-induced damage resulted in severe peritoneal adhesions and a transformation of the muscular layer into collagenous tissue. Intravenous administration of BMSCs and ^APS^BMSCs mitigated the thickening of the peritoneum caused by PDF exposure. IHC analyses revealed a marked increase in collagen protein and fibronectin expression within the peritoneal layers of rats treated with PDF. However, following treatment with BMSCs and ^APS^BMSCs, these expression levels significantly diminished, with ^APS^BMSCs exhibiting more pronounced reparative effects compared to BMSCs alone (Fig. 2B).

Additionally, α-SMA and vimentin mRNA and protein levels were significantly elevated in the PDF group, as evidenced by Western blot and qRT-PCR analyses. These increases were notably mitigated following treatment with BMSCs and ^APS^BMSCs. Moreover, the PDF-induced downregulation of E-cadherin was significantly reversed upon the administration of both BMSCs and ^APS^BMSCs (Fig. 2C-E). These findings indicate that ^APS^BMSCs offer a more pronounced therapeutic benefit in ameliorating PF in rats compared to BMSCs alone.

### APS pretreatment enhances the homing activity of BMSCs in rats

**Fig. 3:**
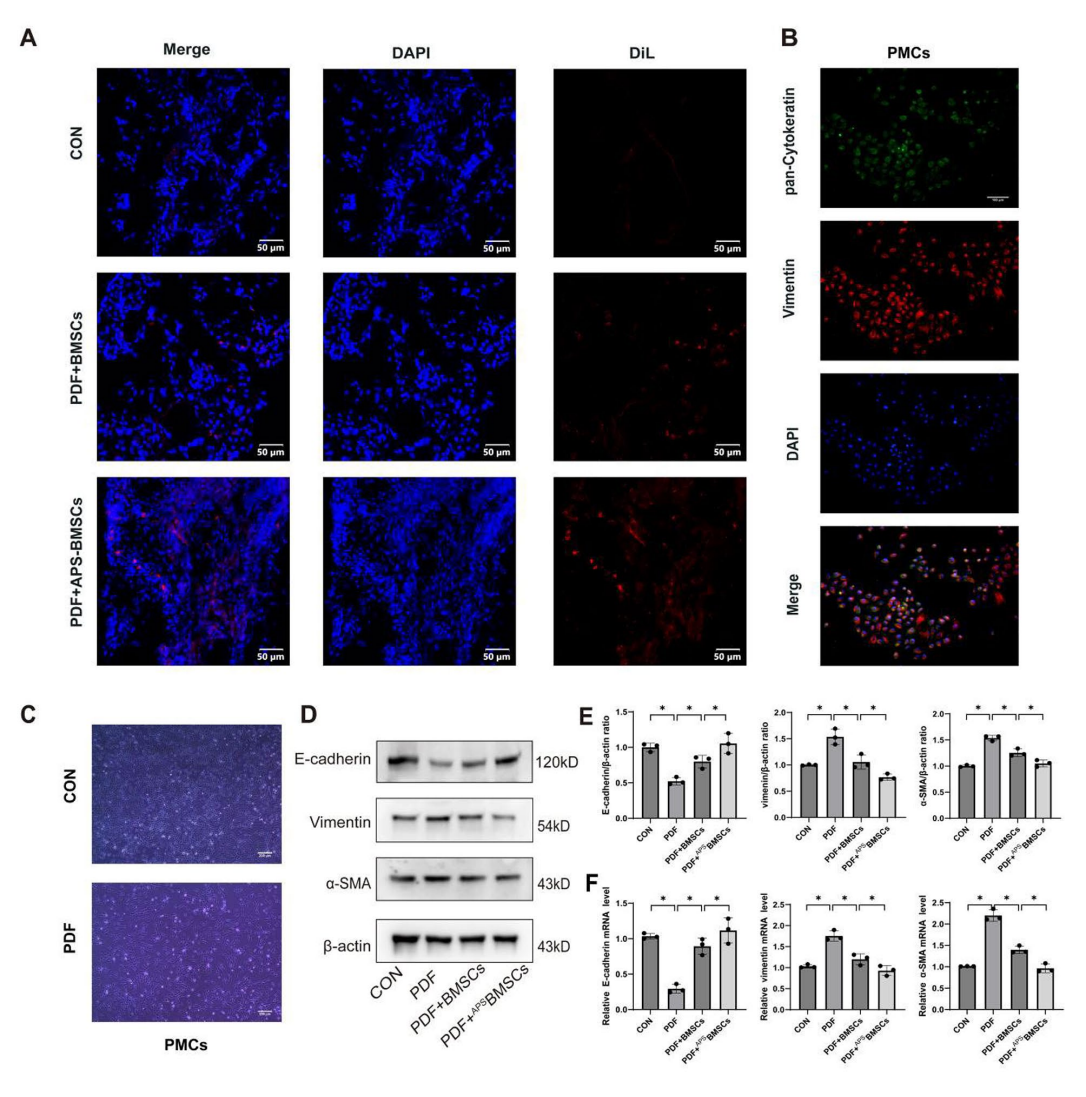
APS promotes the homing of BMSCs and inhibits the MMT of PMC. (**A**) APS pretreatment enhanced the homing activity of BMSCs after injection into rats. After 24 h of transfer, confocal microscopy was used to observe Dil-labeled BMSCs and ^APS^BMSCs in the peritoneum of the control, PDF + BMSCs, and PDF + ^APS^BMSCs groups of rats (scale bar = 50 μm). (**B**) Immunofluorescent staining of pan-cytokeratin and vimentin (100× magnification, scale bar = 100 μm). (**C**) Morphology of PMCs (40× magnification, scale bar = 200 μm). (**D**) Western blot analysis was performed to assess the expression levels of MMT markers in PMCs after PDF treatment and co-culture with BMSCs. (**E**) The quality statistics of Figure D are represented as a histogram. (**F**) qRT-PCR analysis was performed to assess the mRNA expression levels of E-cadherin, vimentin, and α-SMA in PMCs

The effective homing of BMSCs is crucial for their therapeutic effectiveness. To visualize and track the transplanted BMSCs in vivo, they were pre-labeled with Dil prior to transplantation. Twenty-four hours post-transplantation, the distribution and localization of Dil-labeled BMSCs in the rat peritoneum were assessed. In the negative control group, where BMSCs were also injected, only a few BMSCs exhibiting red fluorescence were detected within the peritoneal tissue. Conversely, in the peritoneal tissues of rats in both the PDF + BMSCs and PDF + ^APS^BMSCs groups, a noticeable presence of recruited Dil-labeled BMSCs showing red fluorescence was observed. Notably, the APS-pretreated BMSCs demonstrated more intense red fluorescent signals and a higher abundance in the peritoneal tissue compared to their non-pretreated counterparts (Fig. 3A). This suggests that the enhanced antifibrotic effects observed following APS pretreatment may be due to improved homing capabilities of BMSCs to the peritoneal tissues, thereby facilitating increased interactions with PMCs.

### APS enhances the ability of BMSCs to reverse MMT induced by PDF

To further delve into how APS augments the therapeutic mechanisms of BMSCs in ameliorating PF, we isolated PMCs from rat peritoneal tissue and assessed the impact of BMSCs and ^APS^BMSCs on PDF-induced MMT.

In vitro experiments revealed that PMCs typically exhibited a cobblestone-like growth pattern under optical microscopy. Immunofluorescence analysis verified the presence of vimentin and pan-cadherin, markers indicative of PMC identity. Initially, PMCs underwent treatment with 3% PDF for 24 h to induce MMT. Post-PDF exposure, PMCs displayed an elongated morphology, a decline in the characteristic cobblestone-like cell organization, and transformations reminiscent of fibroblastic changes (Fig. 3B-C).

Western blot analysis was utilized to assess the expression of key proteins, including E-cadherin, α-SMA, and vimentin in PMCs following co-culture experiments. Relative to the control group, the PDF group exhibited a marked increase in α-SMA and vimentin levels. In contrast, both the PDF + ^APS^BMSCs and PDF + BMSCs groups displayed reduced expression of these markers. Notably, the ^APS^BMSCs group demonstrated a more pronounced reduction in α-SMA and vimentin levels compared to the BMSCs alone. Concurrently, E-cadherin expression significantly diminished following PDF treatment. However, while the PDF + ^APS^BMSCs and PDF + BMSCs groups showed an increase in E-cadherin levels, they did not reach statistical significance compared to the control group (Fig. 3D-E). qRT-PCR analysis corroborated these protein expression trends. Specifically, compared to the PDF group, both the PDF + ^APS^BMSCs and PDF + BMSCs groups exhibited decreased levels of α-SMA and vimentin and increased expression of E-cadherin, with ^APS^BMSCs showing superior restoration effects (Fig. 3F). These findings suggest that APS can enhance BMSCs’ capacity to counteract PDF-induced MMT, positioning APS-preconditioned BMSCs as a potentially effective therapeutic strategy for addressing MMT.

### APS pretreatment enhances the migration ability of BMSCs through SDF-1/CXCR4 chemotaxis

**Fig. 4:**
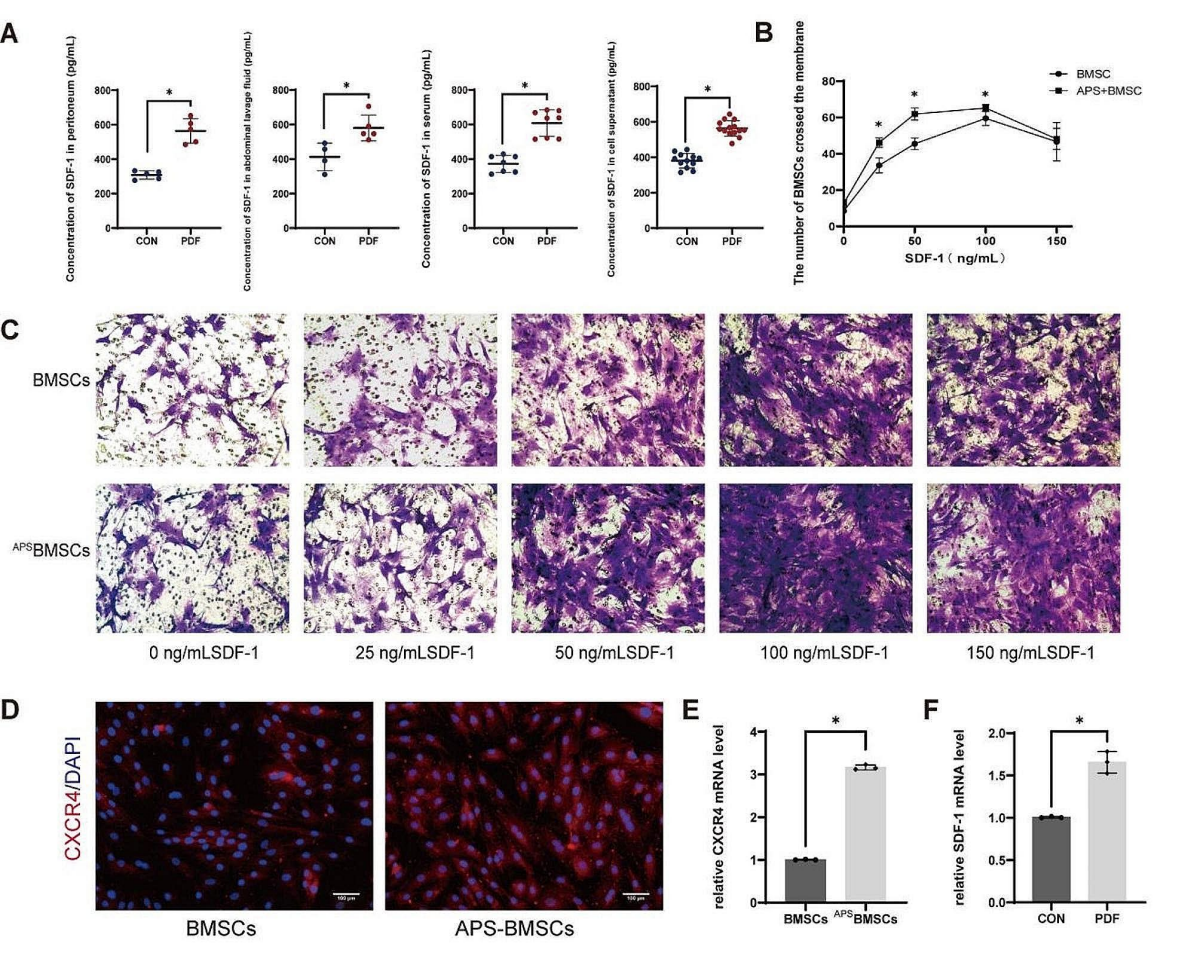
Pre-treatment with APS enhances the migration ability of BMSCs through the SDF-1/CXCR4 chemotactic effect. (**A**) The expression of SDF-1 in the serum, peritoneum, peritoneal lavage fluid, and PMC supernatant was measured using the ELISA method in the control and PDF groups. (**B-C**) Transwell migration assays were conducted with BMSCs and ^APS^BMSCs, using 0, 25, 50, 100, or 150 ng/mL of SDF-1 in vitro (×200) to evaluate their migratory capacities. (**D**) Immunofluorescent staining was performed to confirm the expression of the CXCR4 protein in BMSCs and ^APS^BMSCs (×100). (**E**) qRT-PCR analysis was conducted to determine the expression of CXCR4 mRNA in BMSCs. (**F**) qRT-PCR analysis was performed to evaluate the expression of SDF-1 mRNA in PMCs

The SDF-1/CXCR4 axis is recognized as a critical chemotactic factor/receptor pair facilitating cell homing [12, 22, 23]. To evaluate the effect of APS on the migration capacity of BMSCs mediated by the SDF-1/CXCR4 axis, we conducted the following investigations:

The levels of SDF-1 in the serum, peritoneum, intra-abdominal lavage fluid, and supernatant of PMCs in the control and PDF groups were determined by ELISA. Findings revealed that SDF-1 concentrations were significantly elevated in the PDF group compared to controls (Fig. 4A). This increase suggests an induction of SDF-1 secretion as a response to PD. Additionally, qRT-PCR assays further confirmed a significant elevation of SDF-1 expression in PMCs after PDF exposure compared to the control specimens (Fig. 4F).

To evaluate the impact of SDF-1 on the migratory capability of BMSCs, we performed a transwell migration assay using SDF-1 concentrations ranging from 0 to 150 ng/mL. The chemotactic response of BMSCs increased in a dose-dependent manner when exposed to SDF-1 concentrations from 0 to 100 ng/mL. Notably, BMSCs pre-treated with 1 mg/mL APS exhibited an enhanced migratory response, surpassing that of untreated BMSCs, indicating that APS pre-treatment boosts the chemotactic reaction to SDF-1 (Fig. 4B-C).

Furthermore, to understand the mechanism behind APS’s enhancement of BMSC engraftment efficiency, we assessed the mRNA expression of CXCR4, a key receptor mediating MSC chemotaxis. APS treatment significantly increased CXCR4 mRNA levels in BMSCs. This was corroborated by immunofluorescence analysis, which highlighted the upregulation of CXCR4 in BMSCs following APS treatment (Fig. 4D-E).

**Fig. 5:**
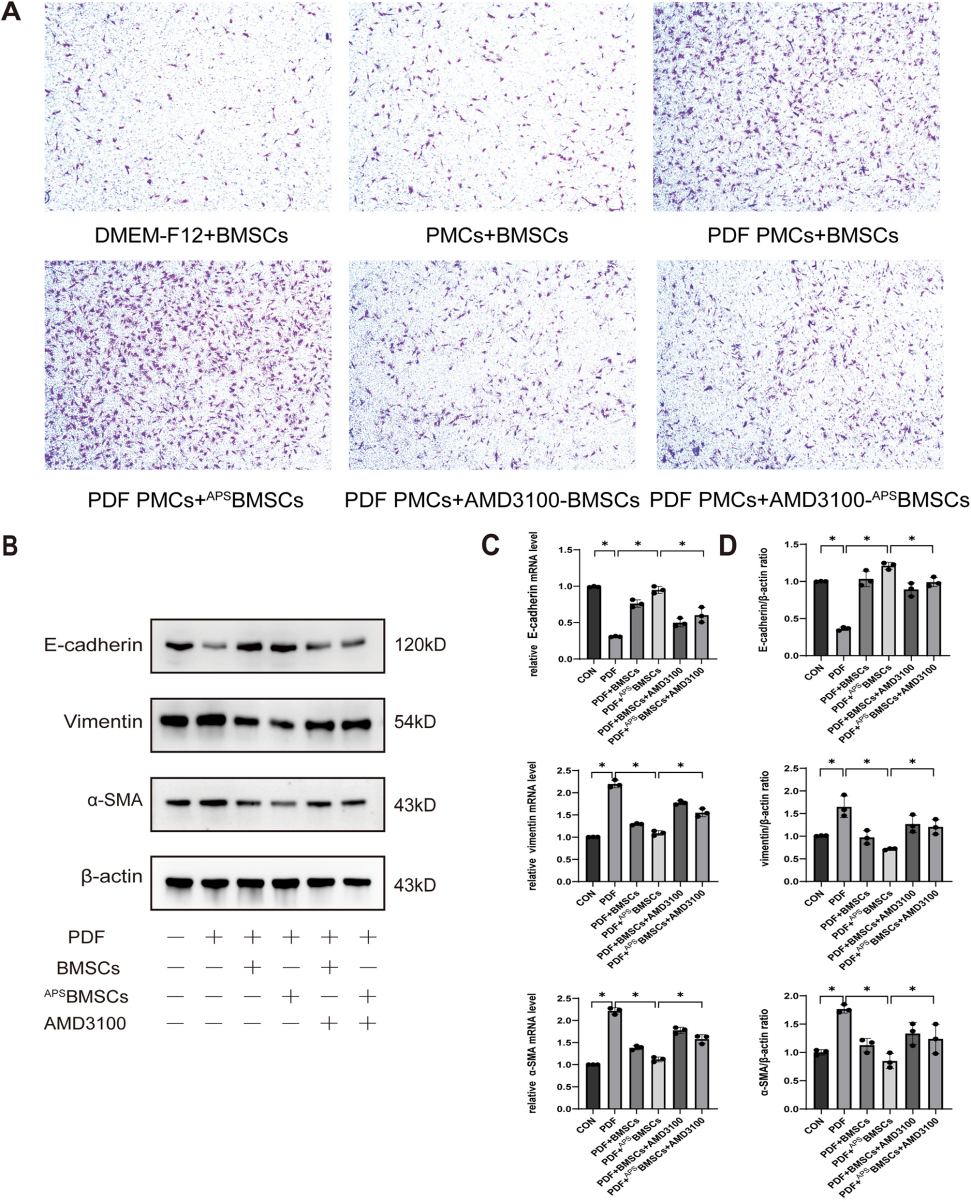
Blocking the SDF-1/CXCR4 axis reduces the migration and therapeutic effects of BMSCs on PDF-induced MMT. (**A**) The cell migration was evaluated using DMEM-F12 + BMSCs, PMCs + BMSCs, PDF PMCs + BMSCs, PDF PMCs + ^APS^BMSCs, PDF PMCs + AMD3100-BMSCs, and PDF PMCs + AMD3100-^APS^BMSCs groups (×40). (**B**) Western blot analysis was conducted to assess the expression of MMT markers in each group. (**C**) The quantitative results of Figure B were represented as a histogram. (**D**) qRT-PCR analysis was performed to determine the expression of E-cadherin, vimentin, and α-SMA mRNA in PMCs

To further elucidate the involvement of the SDF-1/CXCR4 axis in the in vitro migration of BMSCs, we utilized a specific CXCR4 antagonist, AMD3100, in transwell migration assays. After analyzing the in vitro migration of BMSCs induced by 100 ng/mL of SDF-1, we opted to use a concentration of 25 µg/mL of AMD3100 for further investigations (Figure S2). BMSCs and ^APS^BMSCs, either with or without AMD3100 pre-treatment, were placed in the upper chamber of the transwell setup. PMCs, treated with or without PDF, were added into the lower chamber, with DMEM-F12 complete culture solution acting as a negative control. After 24 h, we followed the number of BMSCs that had migrated across the membrane in each experimental condition. The data revealed significant recruitment of BMSCs towards PMCs treated with PDF, with ^APS^BMSCs showing even greater migration than untreated BMSCs. However, the migratory enhancement was notably reduced when the SDF-1/CXCR4 axis was inhibited by AMD3100 (Fig. 5A). These findings suggest that APS pre-treatment considerably boosts the migratory capacity of BMSCs, predominantly through the SDF-1/CXCR4 chemotactic pathway.

### Blocking the SDF-1/CXCR4 axis reduces the therapeutic effect of BMSCs on MMT induced by PDF

To delve deeper into the contribution of the SDF-1/CXCR4 axis to the therapeutic efficacy of BMSCs in treating MMT induced by PDF, this investigation assessed the effects of inhibiting this axis. The expression of E-cadherin, α-SMA, and vimentin was analyzed via Western blot and qRT-PCR (Fig. 5B-C). Relative to the PDF group, the PDF + BMSCs and PDF + ^APS^BMSCs groups exhibited significant reductions in α-SMA and vimentin levels, alongside notable increases in E-cadherin expression. However, upon addition of the CXCR4 antagonist AMD3100 to the PDF + BMSCs and PDF + ^APS^BMSCs groups, there was an observable increase in α-SMA and vimentin levels and a decrease in E-cadherin expression, compared to the groups without AMD3100. These alterations suggest a partial reversal of the therapeutic effects. The PCR findings corroborated the Western blot data (Fig. 5D), indicating that blocking the SDF-1/CXCR4 axis diminishes the healing capabilities of both BMSCs and ^APS^BMSCs. Furthermore, the results highlight that the improved efficacy of BMSCs by APS pre-treatment is significantly mitigated by AMD3100, emphasizing the pivotal role of the SDF-1/CXCR4 axis in mediating the regenerative effects of these cells.

### The effect of APS on the downstream signaling pathway of SDF-1/CXCR4 in BMSCs

**Fig. 6:**
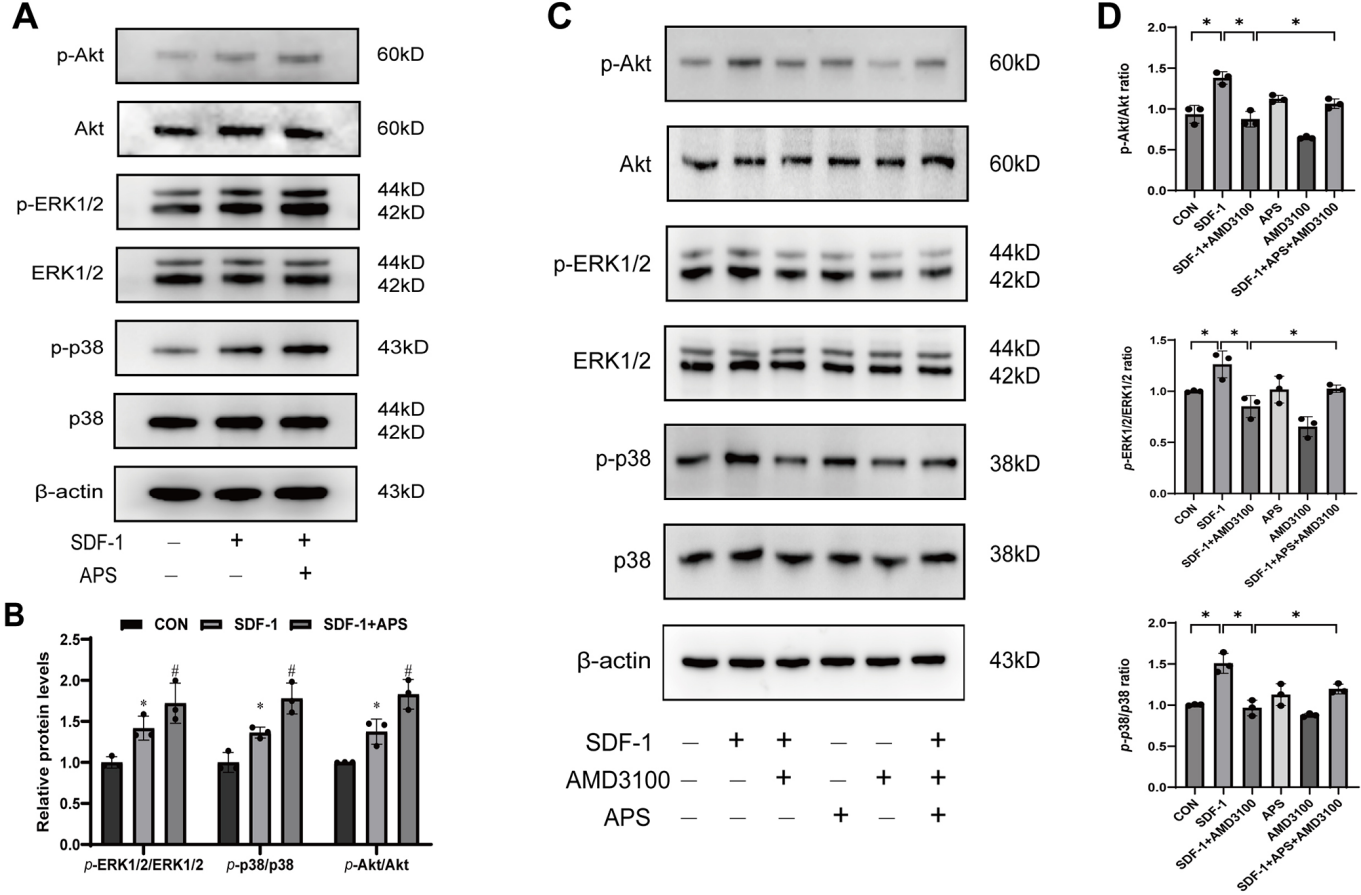
The effect of APS on downstream signaling of SDF-1/CXCR4 in BMSCs. (**A**) Western blot analysis was performed to investigate the effects of SDF-1 and APS on the phosphorylation of ERK1/2, p38, and Akt in BMSCs. (**B**) The ratios of phosphorylated ERK1/2 to total ERK1/2, phosphorylated p38 to total p38, and phosphorylated Akt to total Akt were evaluated. ^*^P < 0. 05 vs. control group; ^#^*P <* 0. 05 vs. SDF-1 group. (**C**) After pre-treating BMSCs with AMD3100 for 1 h, they were subsequently treated with SDF-1 and APS. The levels of phosphorylated ERK1/2, p38, and Akt were analyzed using Western blot. (**D**) The ratios of phosphorylated ERK1/2 to total ERK1/2, phosphorylated p38 to total p38, and phosphorylated Akt to total Akt were assessed

To explore the role of the SDF-1/CXCR4 chemotactic homing mechanism in BMSCs and the modulatory effects of APS, we assessed the activation states of key signaling molecules: ERK1/2, p38, and Akt. Using Western blot analysis, we evaluated the phosphorylation levels of these proteins in BMSCs following stimulation with SDF-1, both with and without APS co-treatment. Our findings indicated that SDF-1 markedly enhanced the phosphorylation of ERK1/2, p38, and Akt, signaling pathways crucial for cell migration and survival. The addition of APS to SDF-1-treated BMSCs resulted in even greater phosphorylation levels of these signaling molecules (Fig. 6A-B). Interestingly, APS alone did not significantly increase the phosphorylation of these pathways. Furthermore, pre-treatment with the CXCR4 antagonist AMD3100 significantly attenuated the SDF-1-induced phosphorylation of ERK1/2, p38, and Akt. However, this attenuation was partially mitigated by the subsequent treatment with APS (Fig. 6C-D). These observations confirm that SDF-1 engagement with CXCR4 triggers the activation of the ERK1/2, p38, and Akt pathways and that APS can enhance this activation in the presence of SDF-1.

## Discussion

Recent investigations have highlighted the potential of MSC transplantation as a viable therapeutic strategy for fibrosis [24]. MSCs have been demonstrated to extend the lifespan of the peritoneum and shield PMCs from the detrimental impacts of PDF [25, 26]. The therapeutic benefits of transplanted MSCs are derived from various mechanisms, including their capacity for differentiation, secretion of diverse growth factors, immunomodulatory factors, and extracellular vesicles. These actions contribute to the inhibition of fibrotic pathways, such as TGF-β1, Wnt/β-catenin, and glucosamine, thereby mitigating the progression of MMT [27–29]. In our study, in vitro co-culture with BMSCs mitigated PDF-induced MMT in PMCs, and in vivo administration via tail vein injection ameliorated PF induced by intraperitoneal injection of PDF in rats. These outcomes corroborate findings from prior research. Notably, we discerned an enhanced therapeutic efficacy following pre-treatment with APS in BMSCs, suggesting a promising augmentation of their therapeutic potential.

The therapeutic success of MSCs is closely tied to their efficiency of transplantation. In 2023, MSCs were reported to ameliorate injury by facilitating mitochondrial transfer, which necessitates an optimal contact range [30]. Consequently, optimizing transplantation strategies and improving transplantation efficiency remain critical issues. The methodology of MSC intervention in PF is currently subject to debate. Although studies have demonstrated that intraperitoneal injection of MSCs aids in the repair of peritoneal mesothelium, no significant aggregation of MSCs has been observed in the damaged peritoneum [31, 32]. Compared to intraperitoneal delivery, intravenous injection of BMSCs results in relocation to the abdominal cavity via the blood circulation, aligning with the distinct targeted homing characteristics of MSCs [33, 34]. A small Phase I clinical trial conducted in 2023 revealed that intravenous infusion of autologous MSCs could diminish the rate of solute transport across the peritoneal membrane in patients, without serious adverse effects [35]. Therefore, intravenous injection appears to be a more physiological and logical approach [36]. It was found that the combination of herbal medicine and stem cell therapy can increase the proportion of donor MSCs in the target organ after intravenous transplantation [37]. In our study, results of fluorescence tracing indicated that APS enhanced the recruitment of intravenously injected BMSCs to the peritoneal region affected by PDF injury. Based on these findings, we propose that the augmented effect of APS pretreatment on BMSCs may be attributed to its enhancement of BMSC homing.

SDF-1 is a pivotal chemokine that plays essential roles in the maintenance, mobilization, and attraction of stem cells, with its expression notably upregulated at injury sites to draw MSCs, which express the CXCR4 receptor, to the damaged regions [38, 39]. Our findings demonstrated that PDF induced an increase in SDF-1 expression in rat peritoneum, peritoneal cavity, peripheral blood, and PMCs. CXCR4 serves as the primary receptor for SDF-1, however, after multiple in vitro passages, the expression of CXCR4 on BMSCs declines, potentially diminishing their therapeutic impact [40, 41]. BMSCs pretreated with APS exhibited heightened surface CXCR4 expression, enhanced sensitivity to SDF-1, and notably improved migration capabilities. Thus, we propose that APS may enhance BMSC migration and homing to PDF-damaged peritoneum through the SDF-1/CXCR4 axis. This aligns with prior research indicating that herbal constituents such as astragalus saponin and epimedoside can activate cell motility-related signal transduction pathways by increasing CXCR4 expression, thereby facilitating MSC migration and boosting therapeutic outcomes [42, 43]. In our experiments, treatment with the CXCR4-specific antagonist AMD3100 significantly reduced APS’s ability to enhance BMSC migration to damaged PMCs and the subsequent inhibitory effects of BMSCs on MMT. This evidence further supports the notion that APS enhances the repair function of BMSCs by upregulating the expression of CXCR4, promoting their directed migration and homing to the site of injury.

The interaction between SDF-1 and CXCR4 triggers the activation of downstream signaling pathways, including MAPK and Akt, which are pivotal in regulating cell homing, migration, apoptosis, and gene expression [44–46]. A 2022 study demonstrated that using the CXCR4 blocker AMD3100, indirectly suppressed the PI3K/Akt signaling pathway and significantly reduced the recruitment of transplanted MSCs to the ovary [47]. Furthermore, botanicals (e.g. Icariin) have been reported to enhance BMSC migration by augmenting the formation of actin stress fibers through the MAPK signaling pathway [48]. In our experiments, in the presence of SDF-1, APS could further amplify the phosphorylation levels of downstream efectors such as Akt, ERK1/2, and p38, while effectively counteracting the inhibitory effect of AMD3100 on downstream phosphorylation. This enhancement is likely mediated by an increase in CXCR4 expression on the surface of BMSCs.

Although this study illustrates the enhancing effects of APS on BMSCs in an animal model, it does not delve into the specific mechanisms by which BMSCs inhibit PF. Furthermore, a discrepancy exists between cellular migration and in vivo recruitment, suggesting that a more comprehensive experimental design involving the overexpression or knockdown of CXCR4 in vivo would provide deeper insights. Additionally, further investigations are required to ascertain the dose-response relationship, long-term effects, and safety profile of APS. Given the variances between animal models and human conditions, the effectiveness, safety, tissue specificity, and optimal dosages of MSCs in human patients necessitate further exploration. These initiatives are crucial for advancing our understanding of the roles of APS and MSCs in treating fibrotic diseases.

**Fig. 7:**
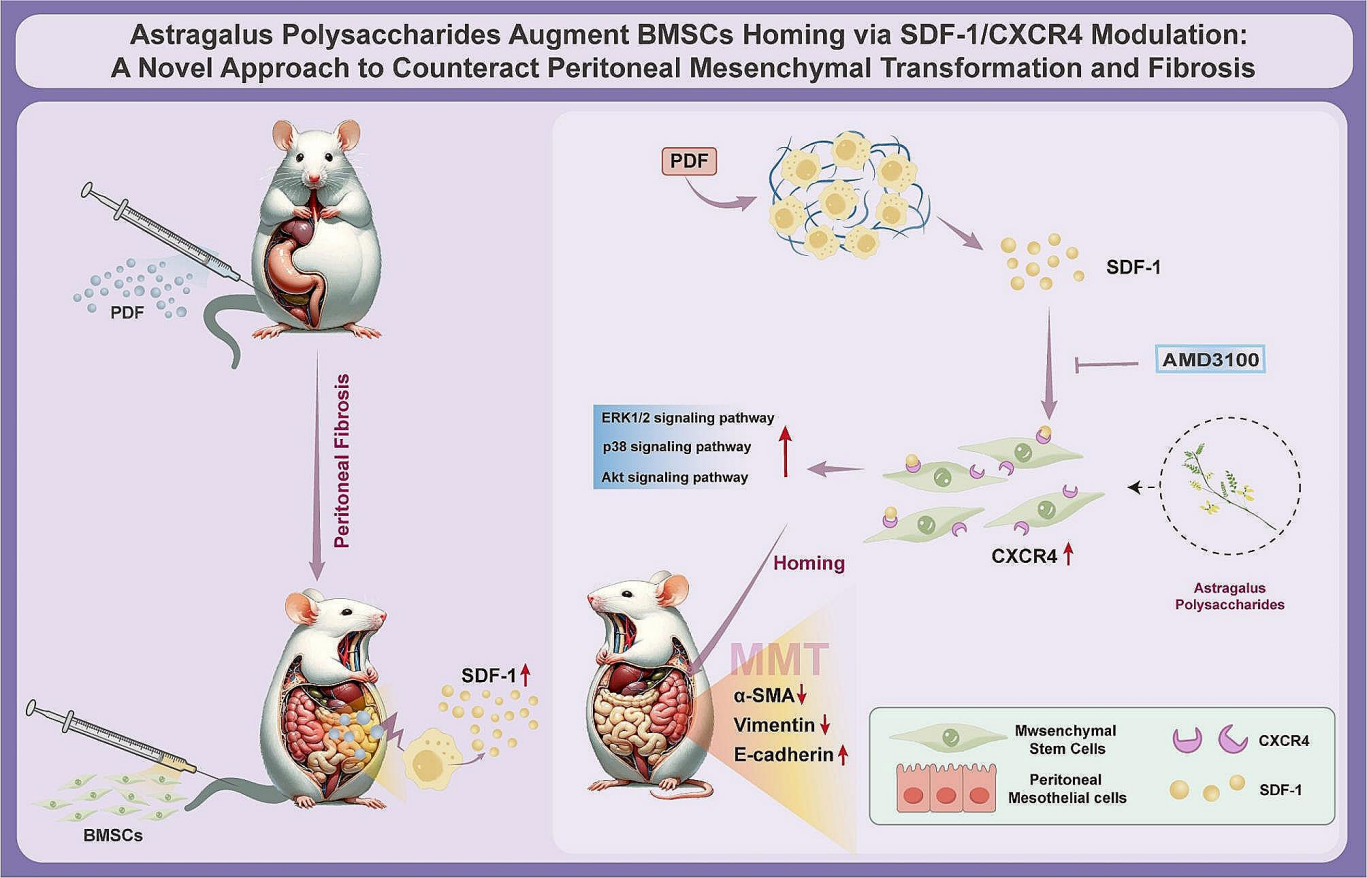
Astragalus Polysaccharides Augment BMSC Homing via SDF-1/CXCR4 Modulation

This study elucidates that APS facilitates the targeted homing of BMSCs via the SDF-1/CXCR4 axis, thereby amplifying the anti-fibrotic efficacy of BMSCs. This underscores a novel therapeutic approach for PF. Additionally, our findings corroborate the effective role of plant-derived drugs, such as APS, in modulating stem cell functionality. This opens new avenues for the integration of botanical compounds in the management and treatment of fibrotic diseases (Fig. 7).

## Conclusion

In conclusion, our research verified that APS could augment the migration and homing capabilities of BMSCs through the upregulation of CXCR4 expression, thereby amplifying their anti-fibrotic effects. This suggests that botanical components capable of enhancing MSC recruitment to damaged areas might offer novel therapeutic avenues for the management of fibrosis.

### Electronic supplementary material

Below is the link to the electronic supplementary material.

Supplementary Material 1

Supplementary Material 2

Supplementary Material 3

## Data Availability

All data generated or analysed during this study are included in this published article and its supplementary information files.

## Abbreviations

APS: Astragalus polysaccharide
BMSCs: bone marrow mesenchymal stromal cells
PDF: peritoneal dialysis fluid
MMT: mesothelial-mesenchymal transition
SDF-1: stromal cell-derived factor-1
CXCR4: chemokine receptor type 4
PD: Peritoneal dialysis
PF: Peritoneal fibrosis
PMCs: Peritoneal mesothelial cells
PBS: Phosphate-buffered saline
FBS: Phosphate-buffered saline
SD: Sprague-Dawley
α-SMA: α-smooth muscle actin
ELISA: Enzyme-linked immunosorbent assay
HE: hematoxylin-eosin
IHC: immunohistochemical
