# Supplementary material for: Astragalus polysaccharides augment BMSC homing via SDF-1/CXCR4 modulation: a novel approach to counteract peritoneal mesenchymal transformation and fibrosis
Source: BMC Complement Med Ther. 2024 May 24;24:204. doi: 10.1186/s12906-024-04483-5 (PMC11127382; doi:10.1186/s12906-024-04483-5)
Supplement: Supplementary file 1 — Supplementary Material 1 [file 12906_2024_4483_MOESM1_ESM.docx]

**Figure S1. Effects of different concentrations of astragalus polysaccharide on the proliferative activity of BMSCs**

The basis for choosing a 0-4 mg/mL concentration range of Astragalus polysaccharides (APS) for testing cell proliferation activity may include the following points: Based on preliminary experimental results or literature review (PMID: 34699323; PMID: 35691200; PMID: 35877777; PMID: 31154455; PMID: 37532241), the cell proliferation activity of APS has been explored at concentrations as low as 50 μg/mL to as high as 400 μg/mL, but not fully covering beyond 1 mg/mL. We also consider that different cell types may have varying sensitivities to APS. Therefore, this study has expanded the concentration range to more comprehensively plot the dose-response curve and determine the highest effective concentration of APS.

**Figure S2. Effects of AMD3100 on the migration of BMSCs**

The experimental basis for selecting concentrations of 25, 50, and 100 μg/mL of AMD3100 to test its effect on the migration of bone marrow stromal stem cells is based on published literature. Specifically, PMID:4737461 provides data on the effect of 25 μg/mL AMD3100 on cell migration inhibition.

The experimental dose and intervention time of PDF were referred to the previous study of our group.PMID: 37437794
