# Supplementary figures and images for: Astragalus polysaccharides augment BMSC homing via SDF-1/CXCR4 modulation: a novel approach to counteract peritoneal mesenchymal transformation and fibrosis
Source: BMC Complement Med Ther. 2024 May 24;24:204. doi: 10.1186/s12906-024-04483-5 (PMC11127382; doi:10.1186/s12906-024-04483-5)

C

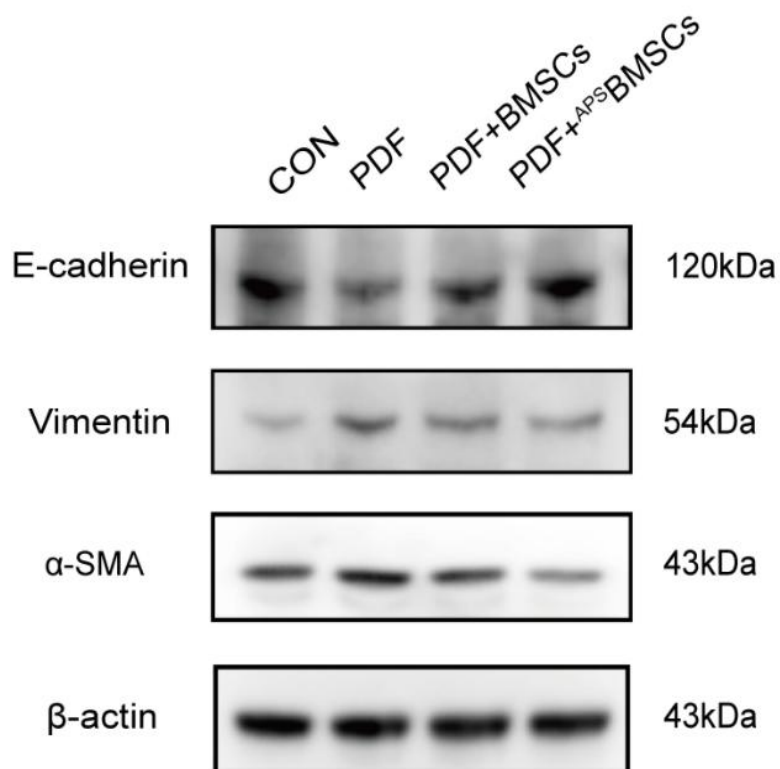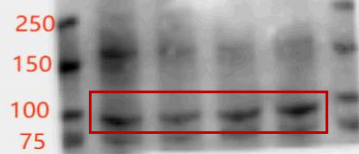

Figure 2C E-cadherin

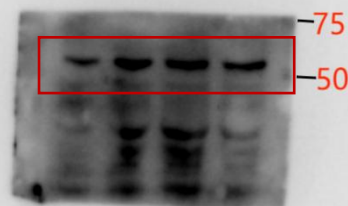

Figure 2C vimentin

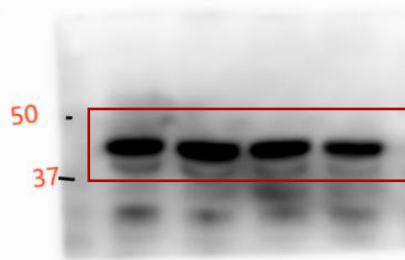

Figure 2C α-SMA

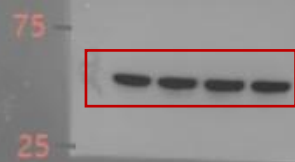

Figure 2C β-actin

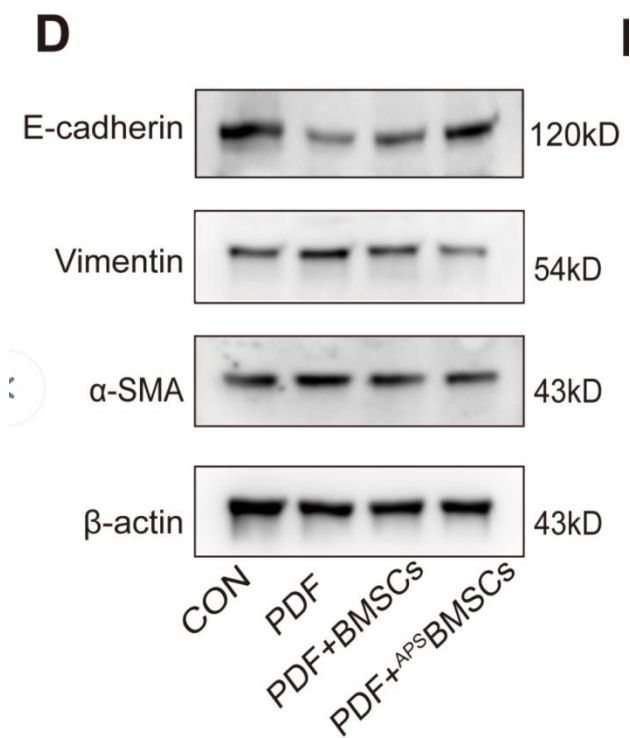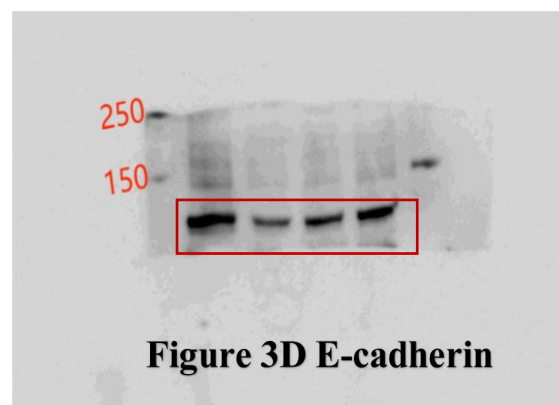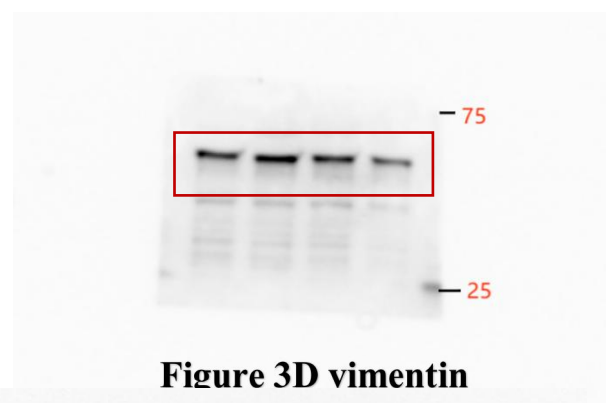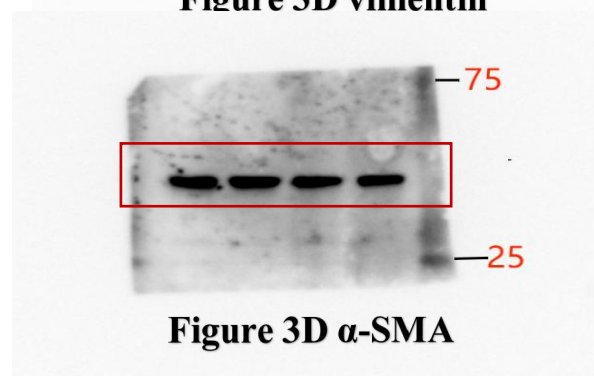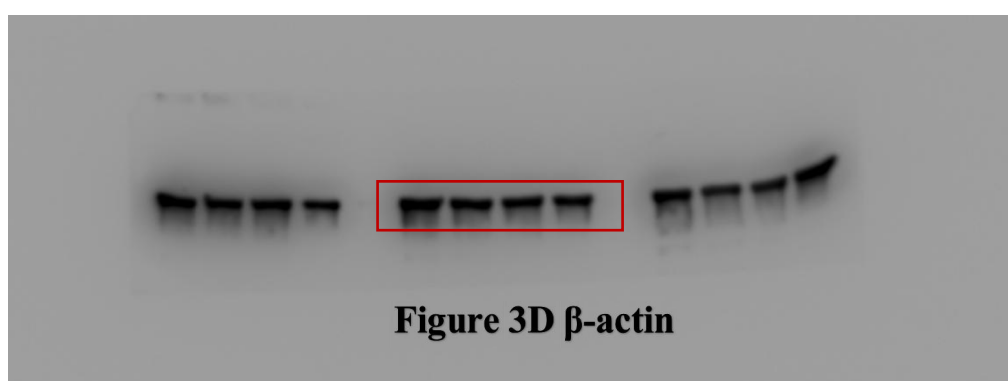

**B**

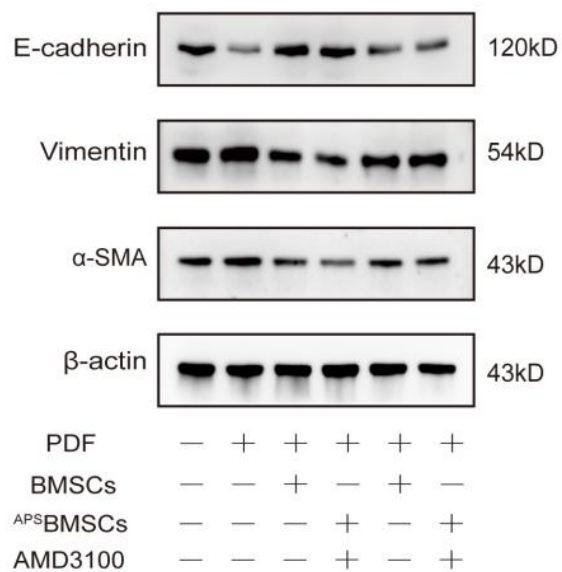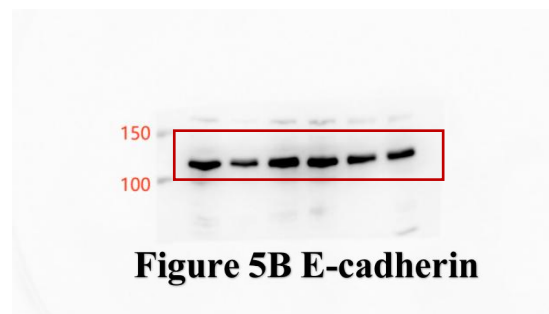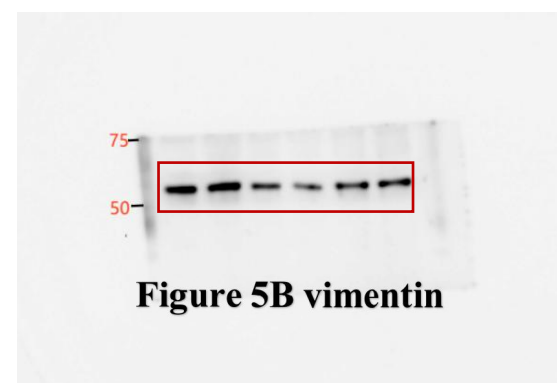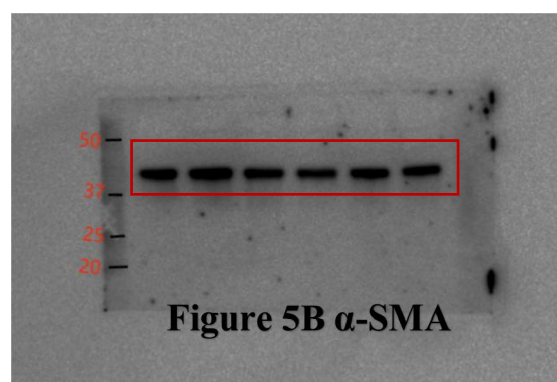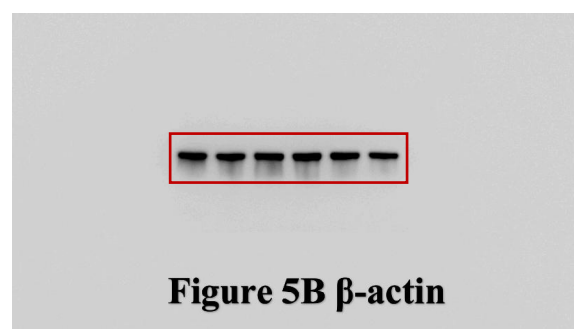

**A**

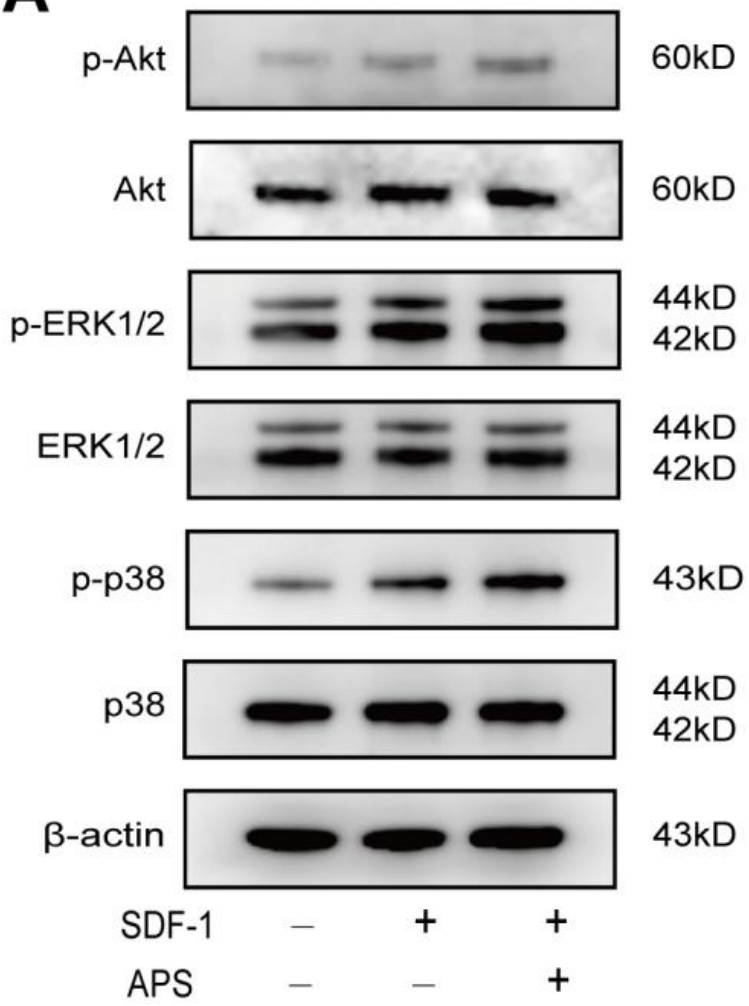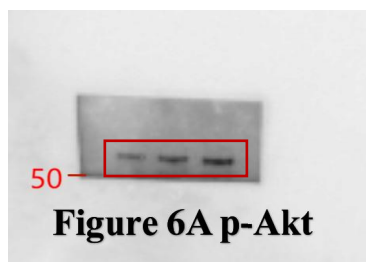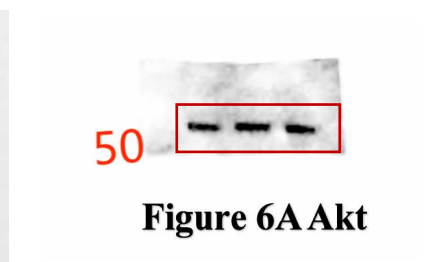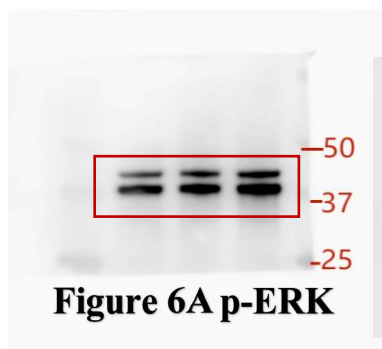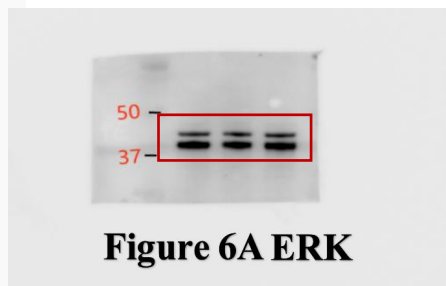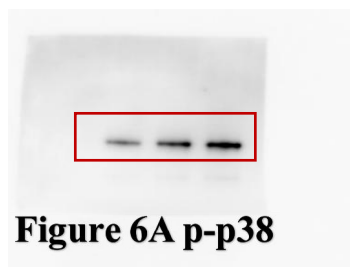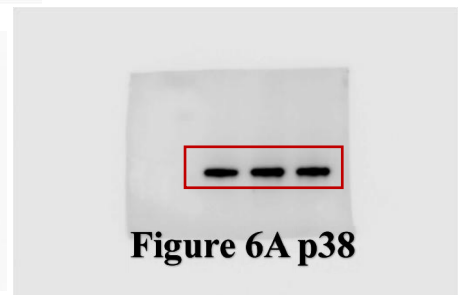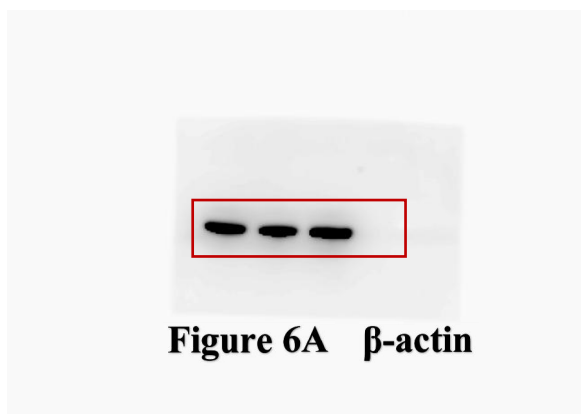

**C**

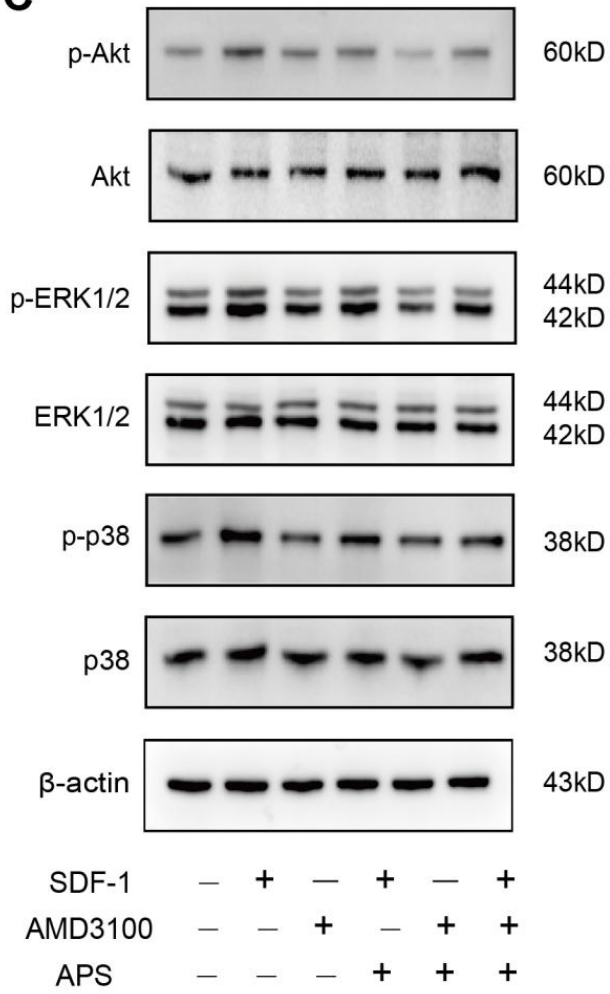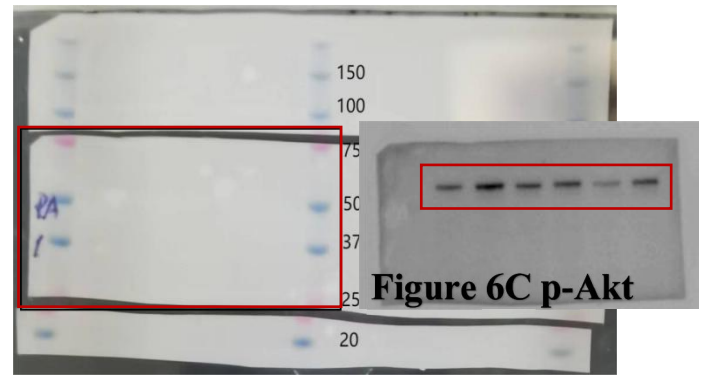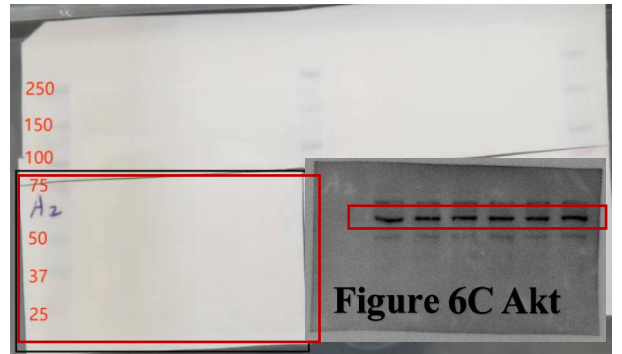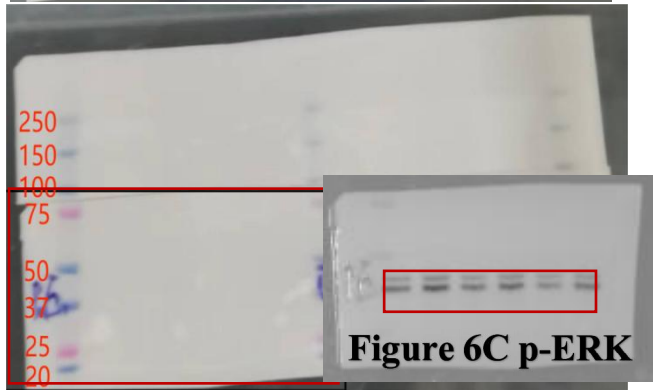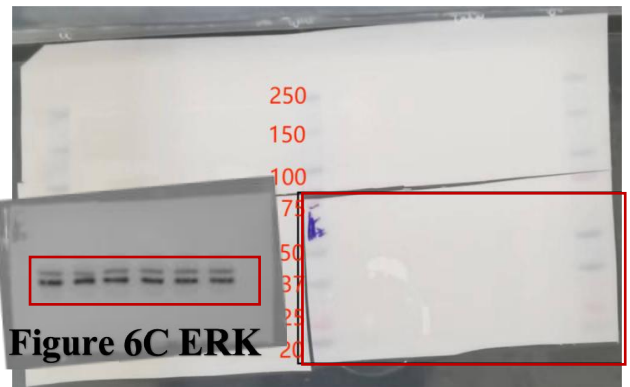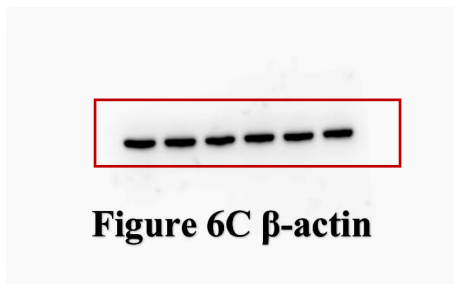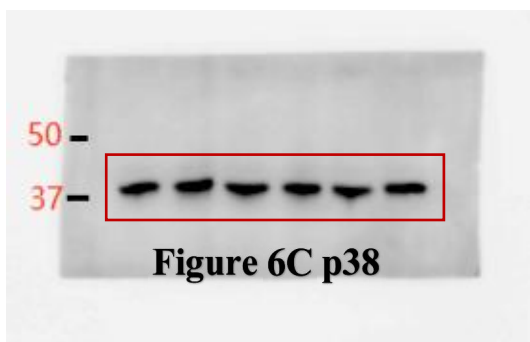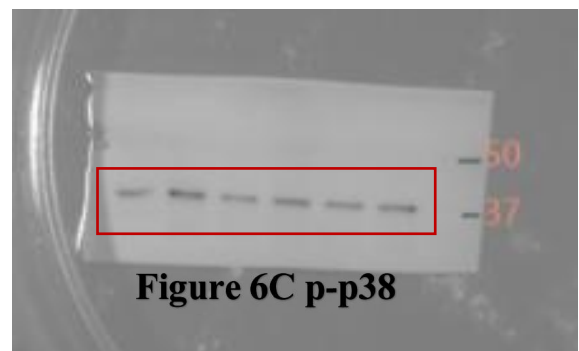

Supplement: Supplementary file 2 — Supplementary Material 2 [file 12906_2024_4483_MOESM2_ESM.pdf]
