# Supplementary material for: Astragalus polysaccharides augment BMSC homing via SDF-1/CXCR4 modulation: a novel approach to counteract peritoneal mesenchymal transformation and fibrosis
Source: BMC Complement Med Ther. 2024 May 24;24:204. doi: 10.1186/s12906-024-04483-5 (PMC11127382; doi:10.1186/s12906-024-04483-5)

# Western Blot

# F2 β-actin

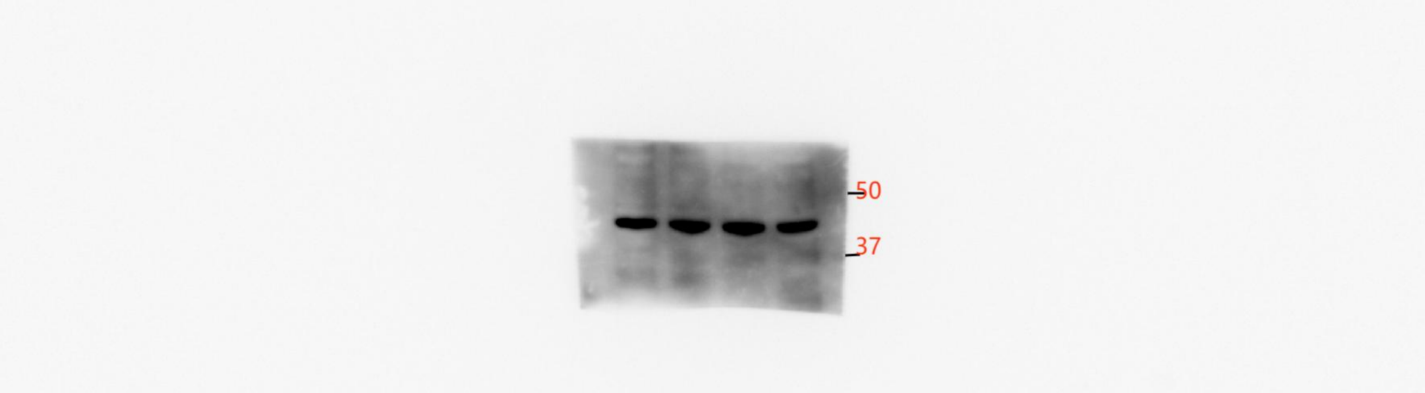


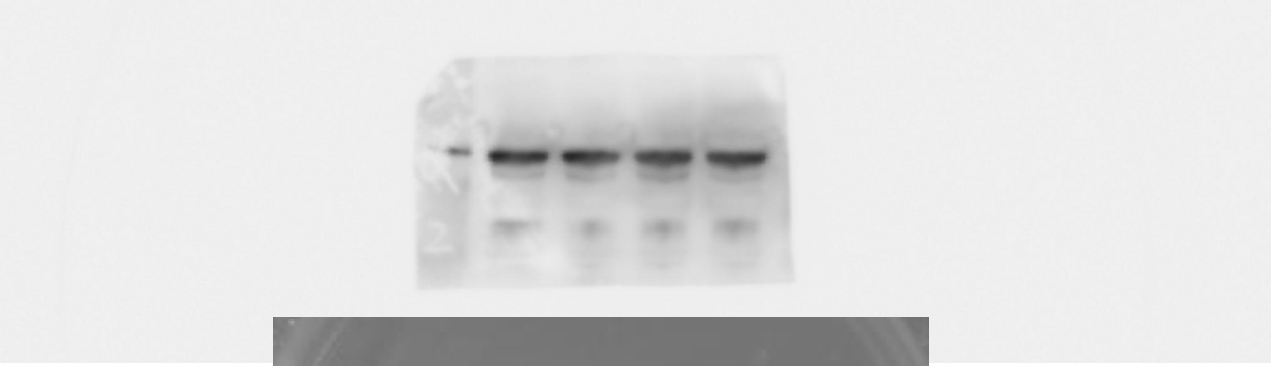

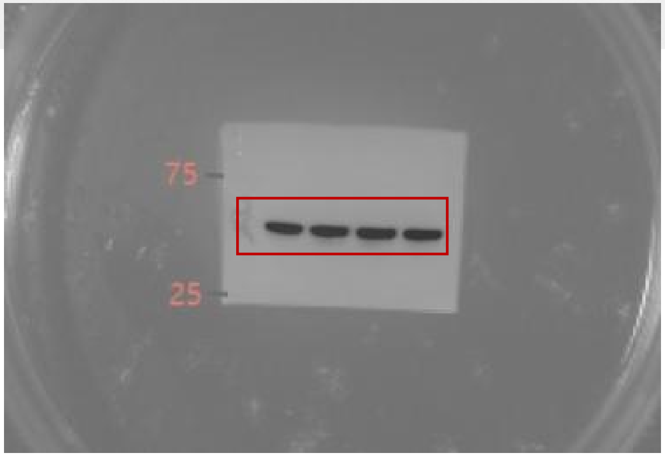

# Figure 2C E-cadherin

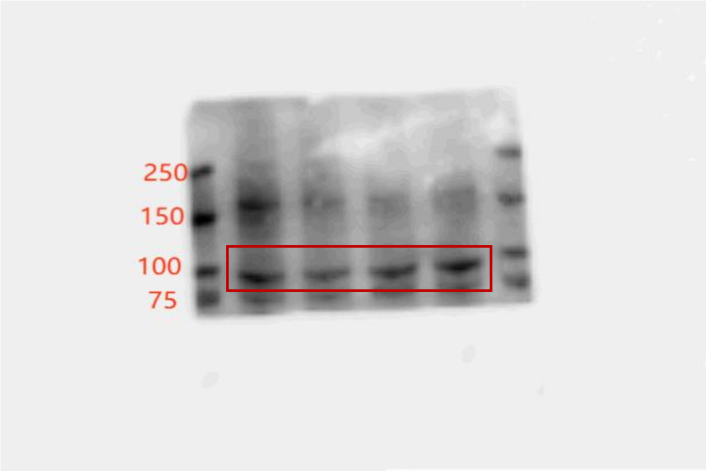


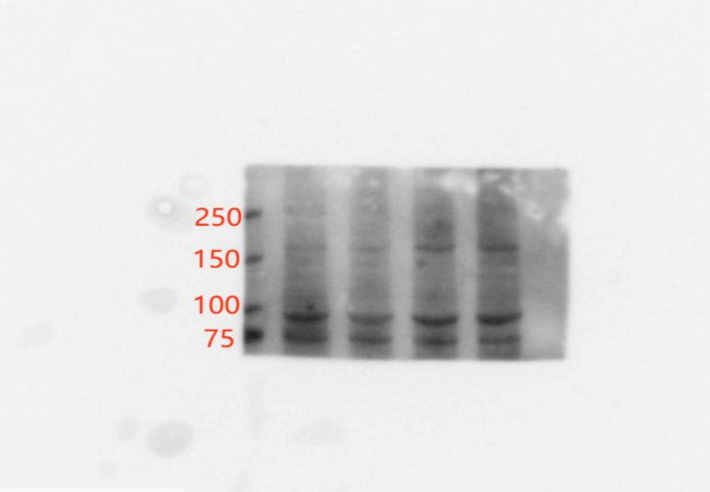


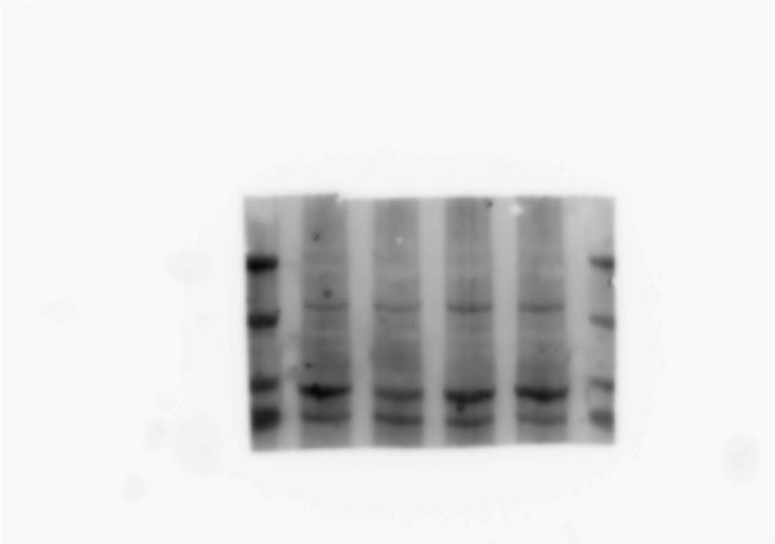

# Figure 2C α-SMA

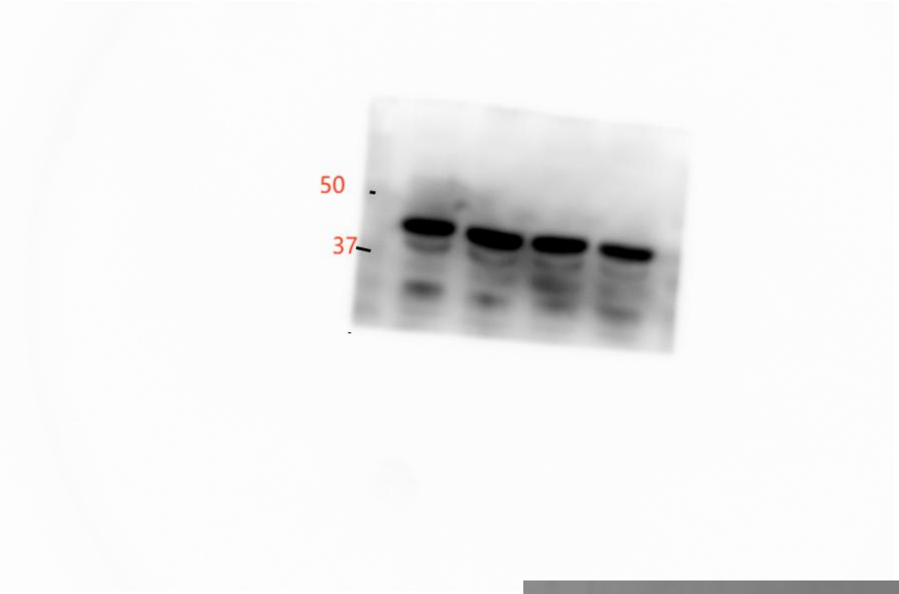


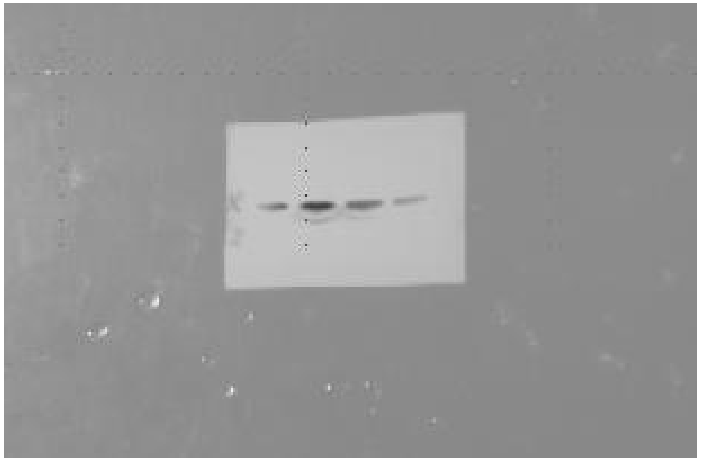

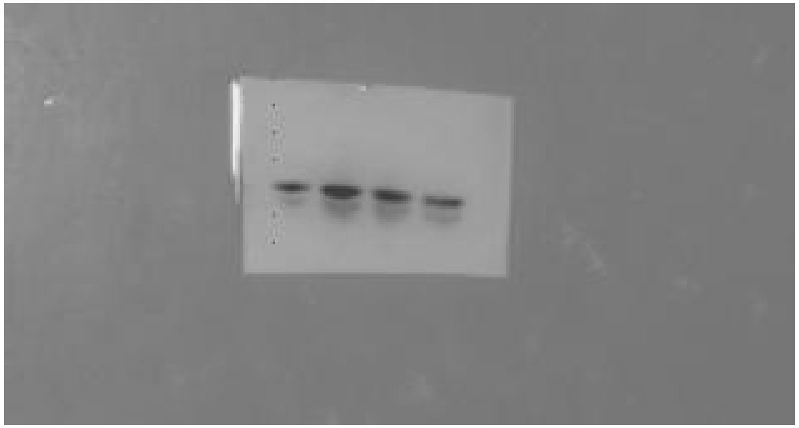

# Figure 2C vimentin

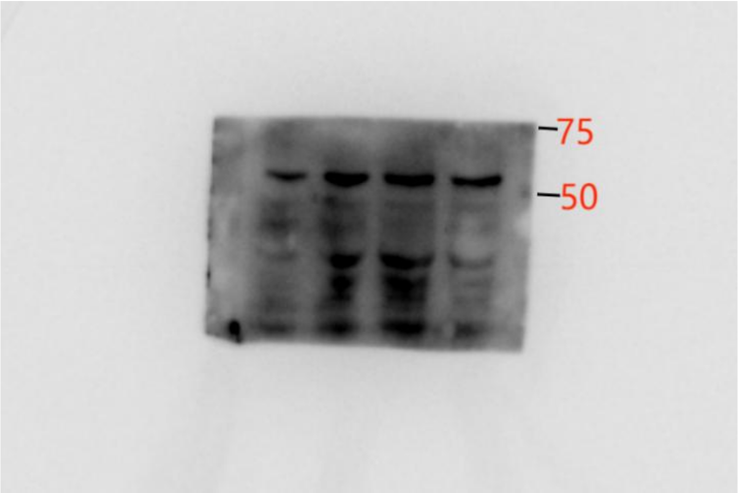


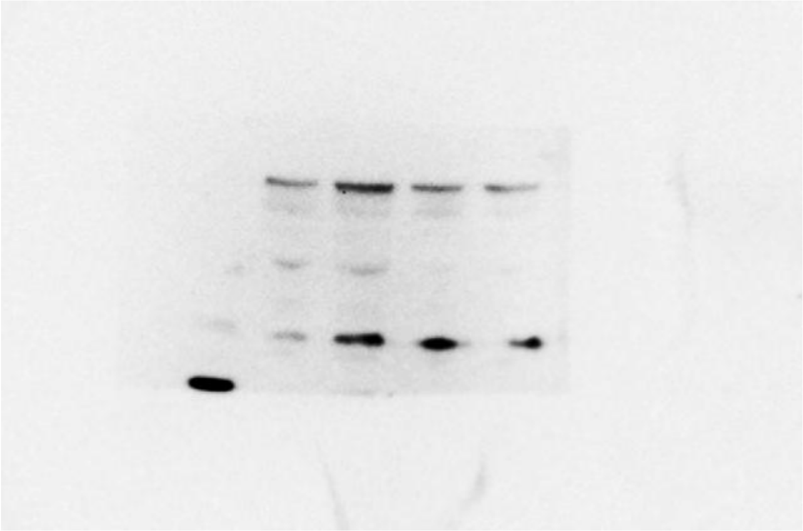

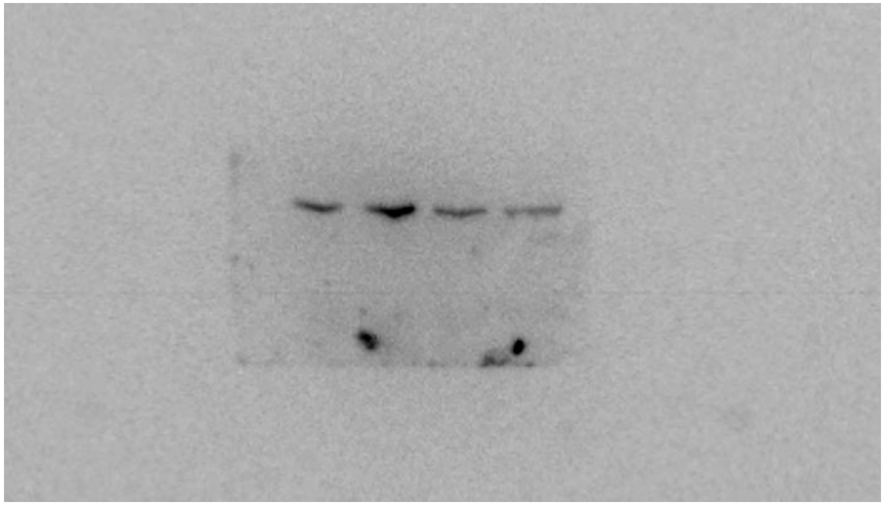

# Figure 3D β-actin

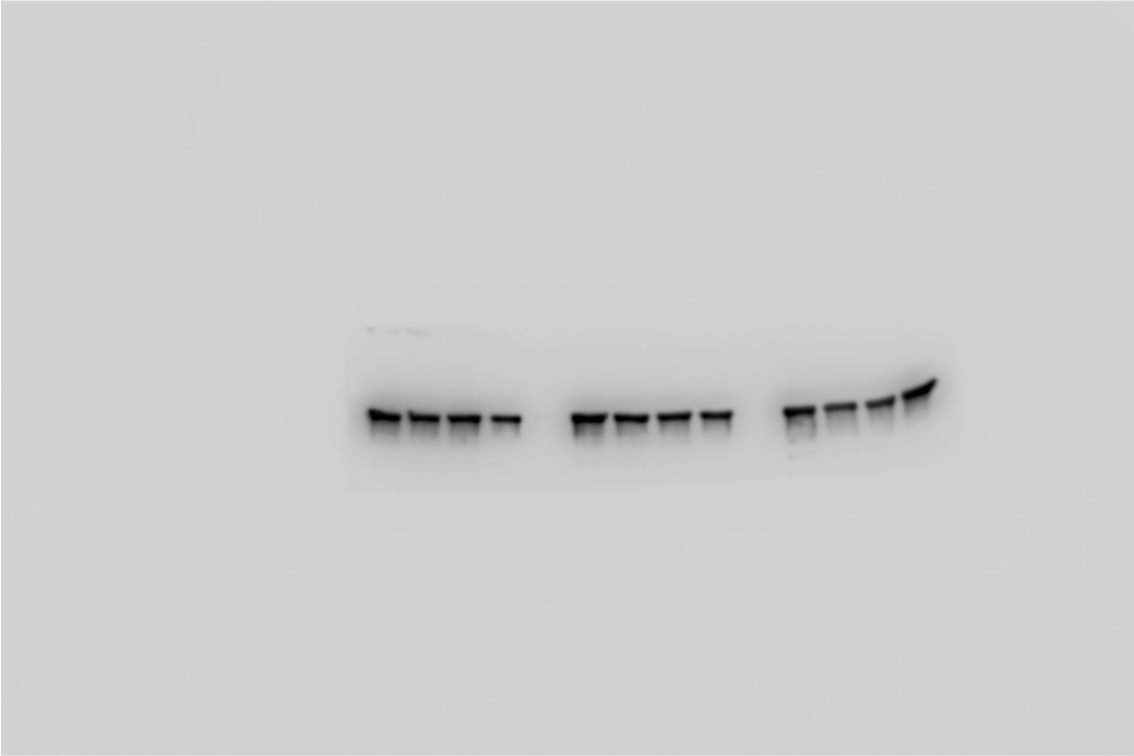

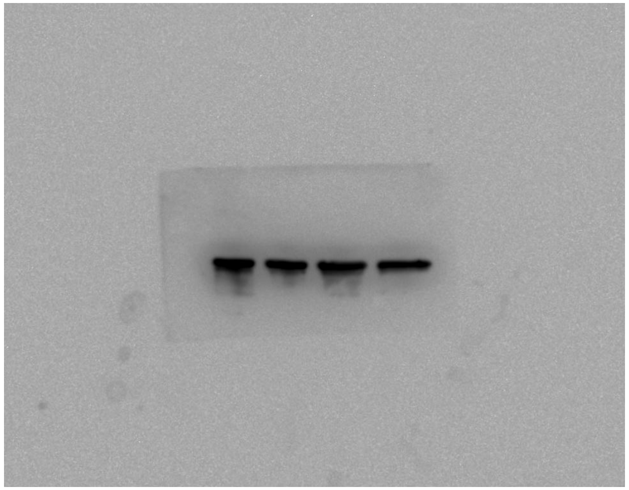

# Figure 3D E-cadherin

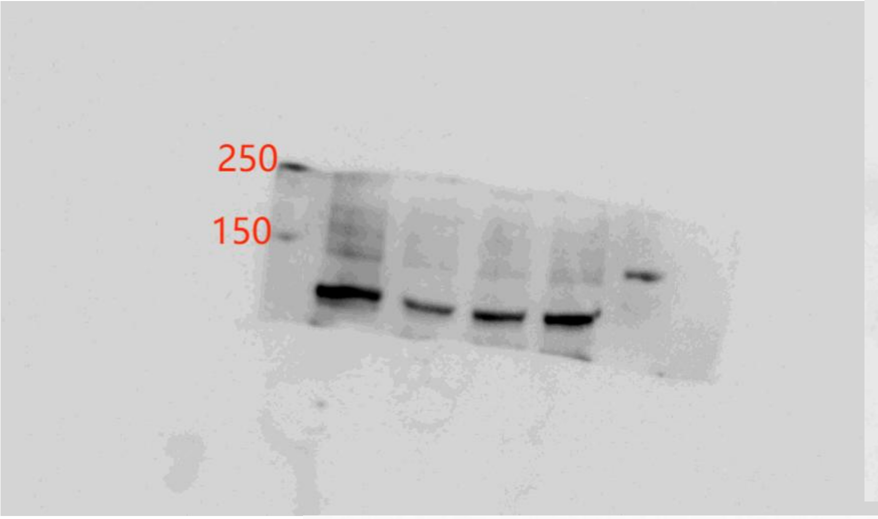


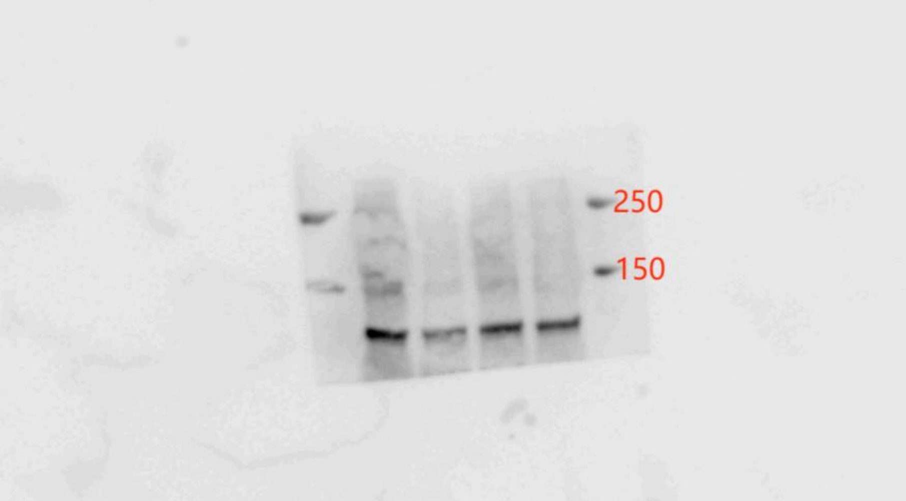


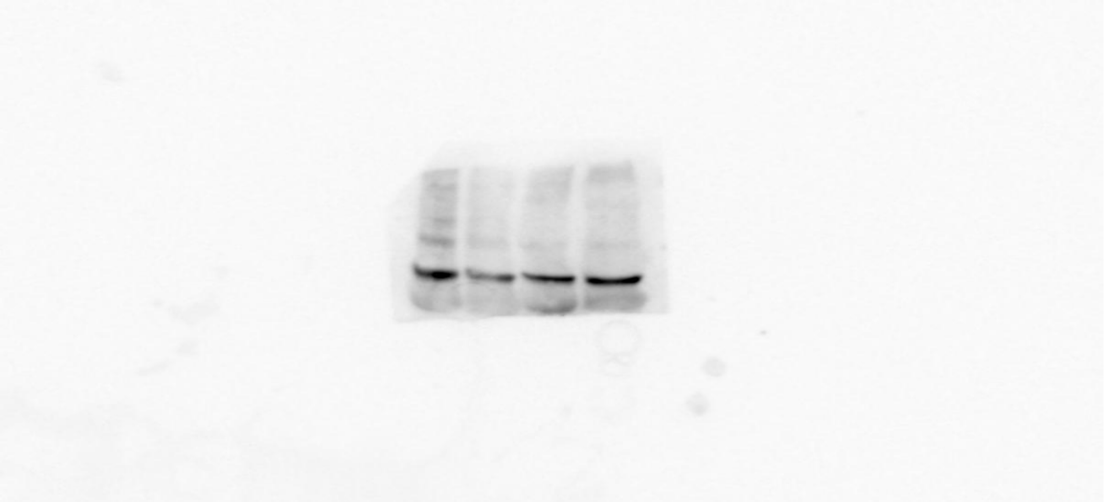

# Figure 3D vimentin

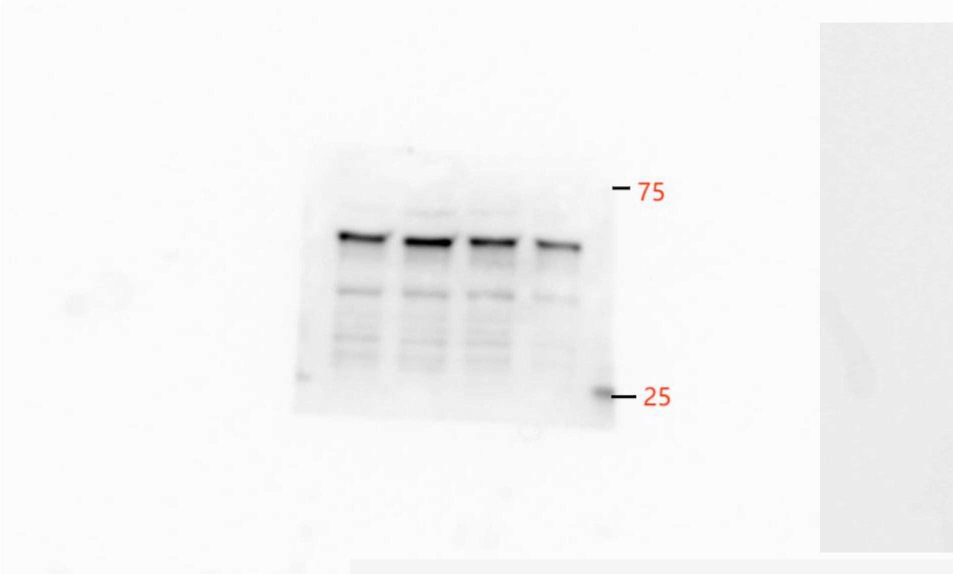


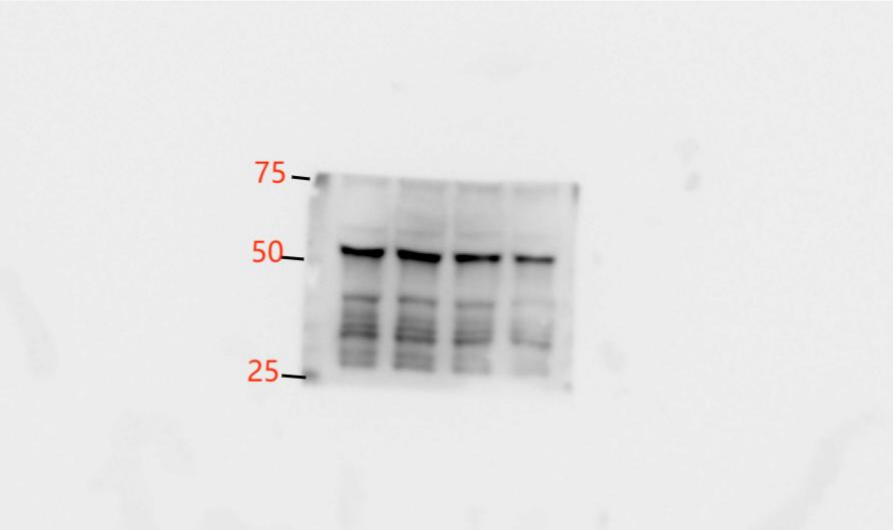


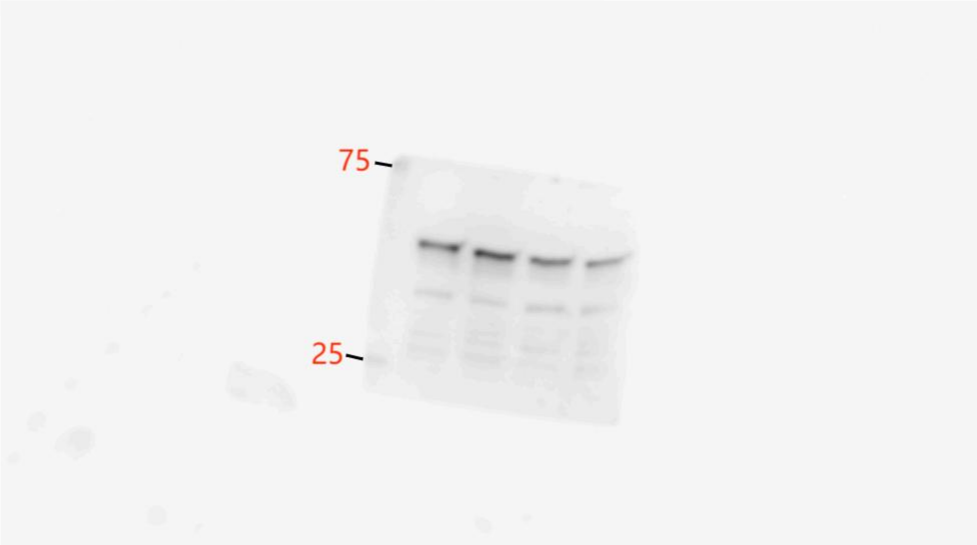

# Figure 3D α-SMA

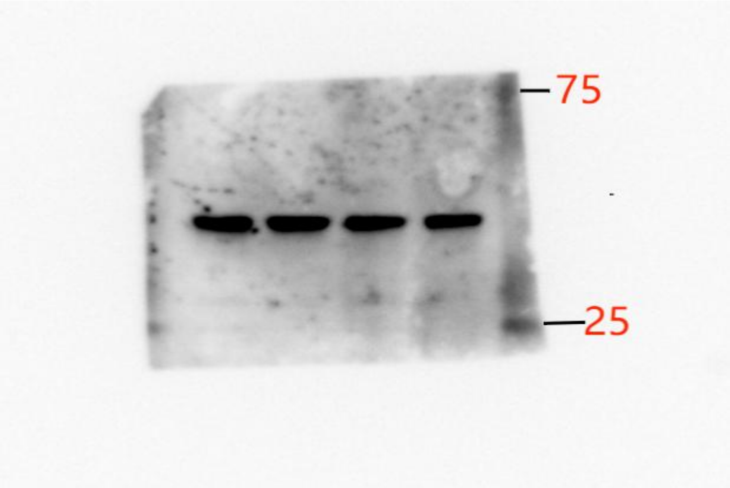


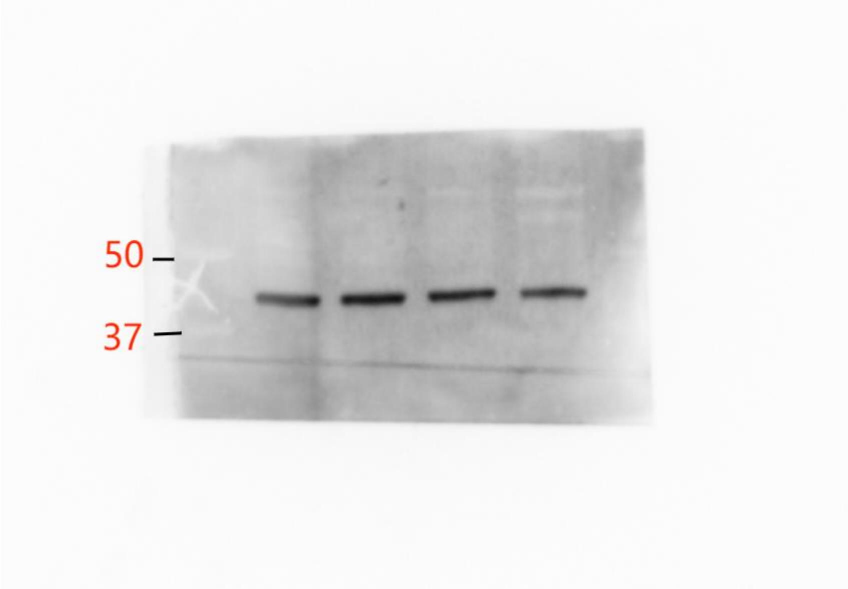


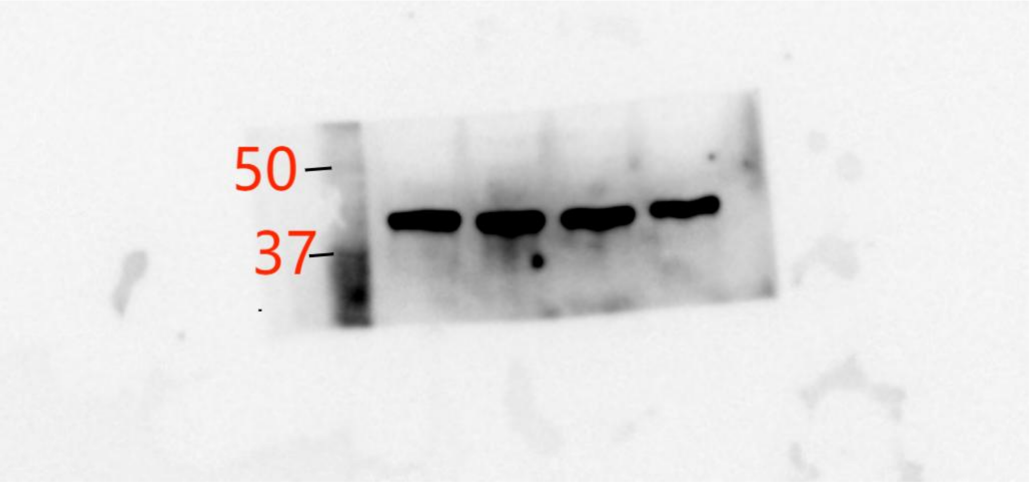

# Figure 5B β-actin

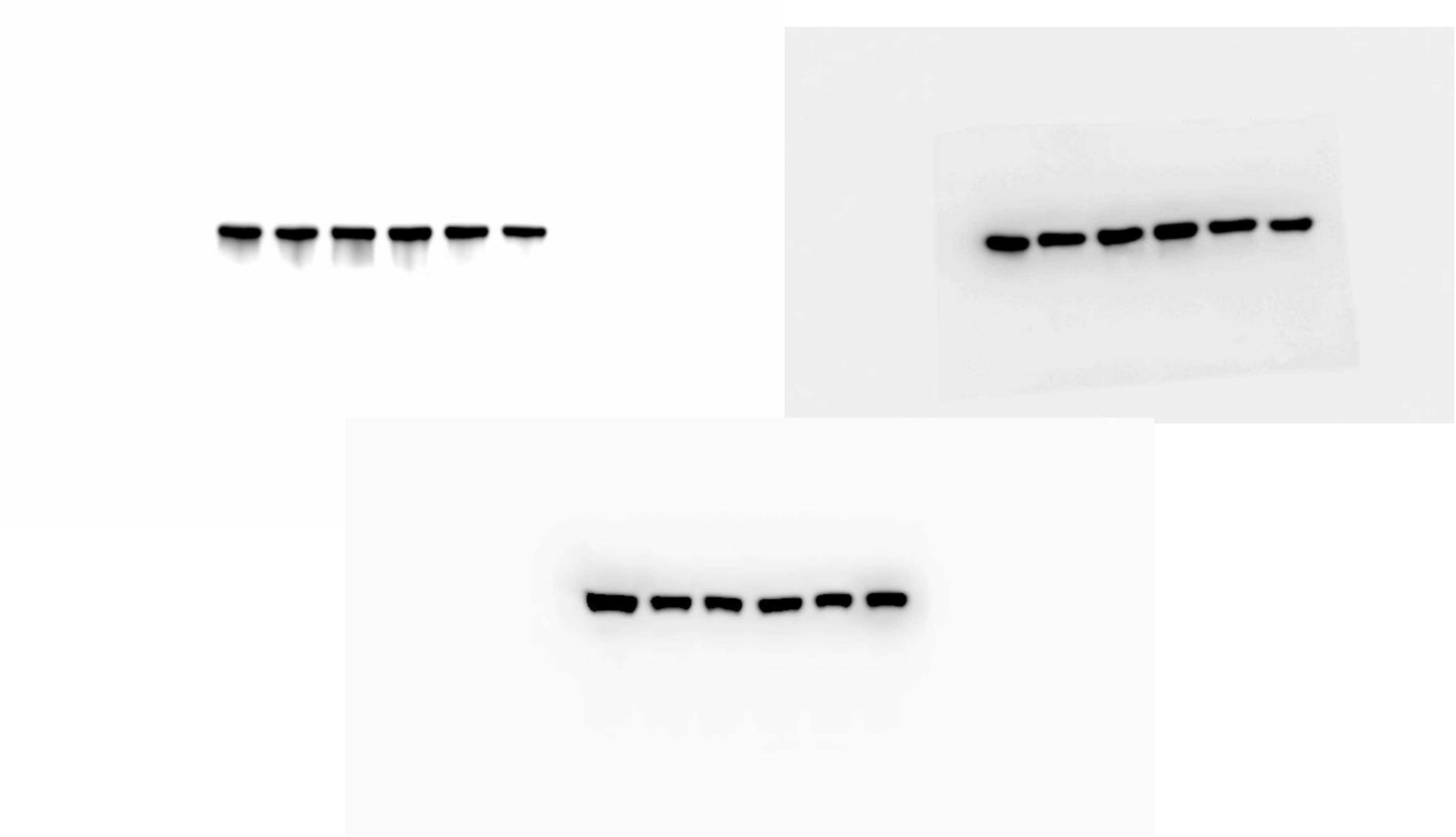

# Figure 5B E-cadherin

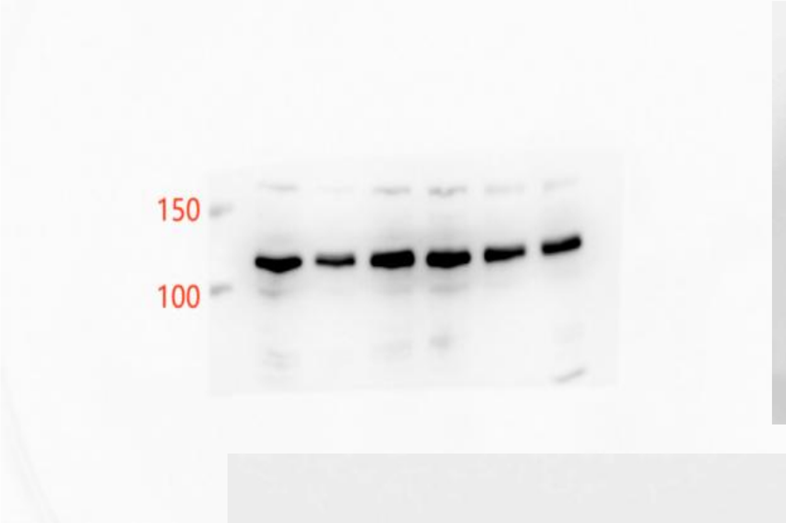


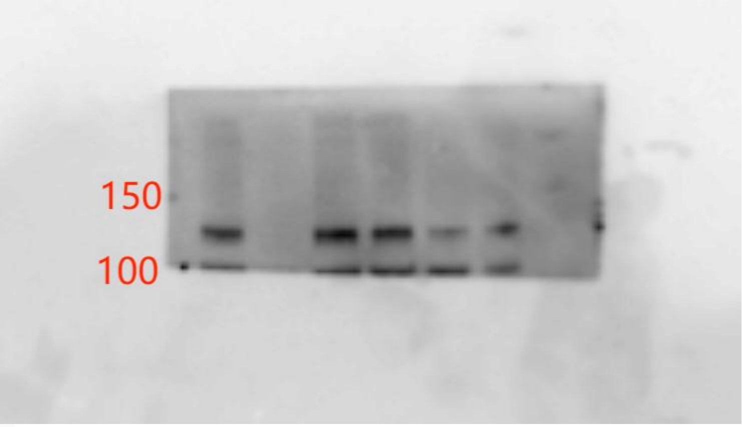


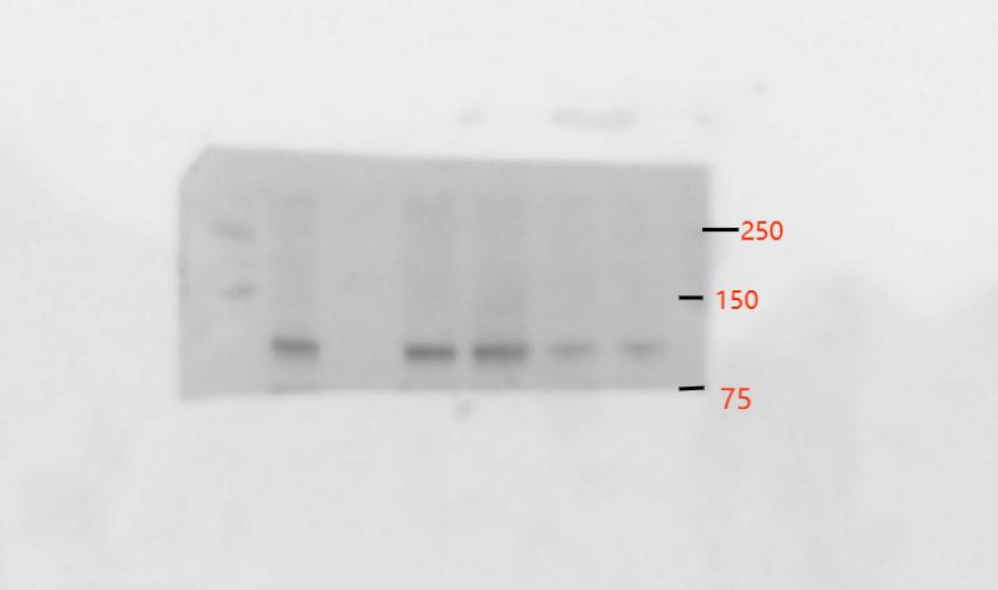

# Figure 5B vimentin

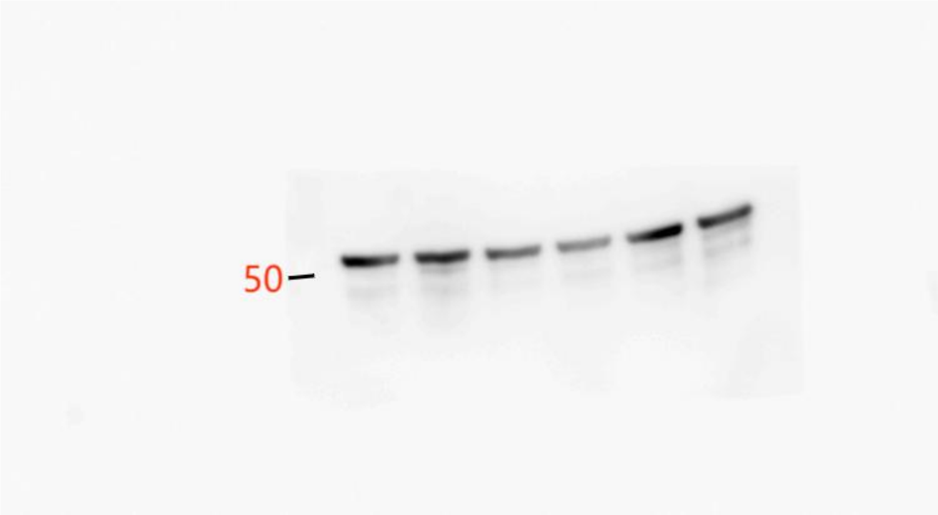


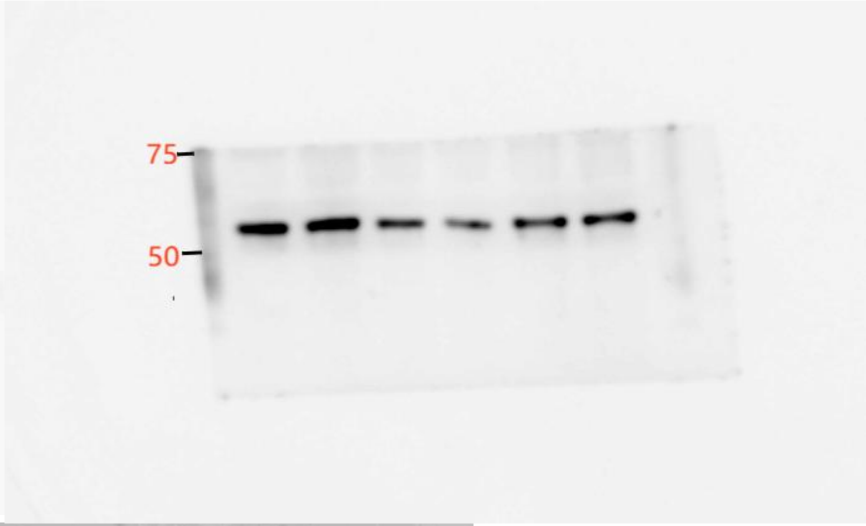


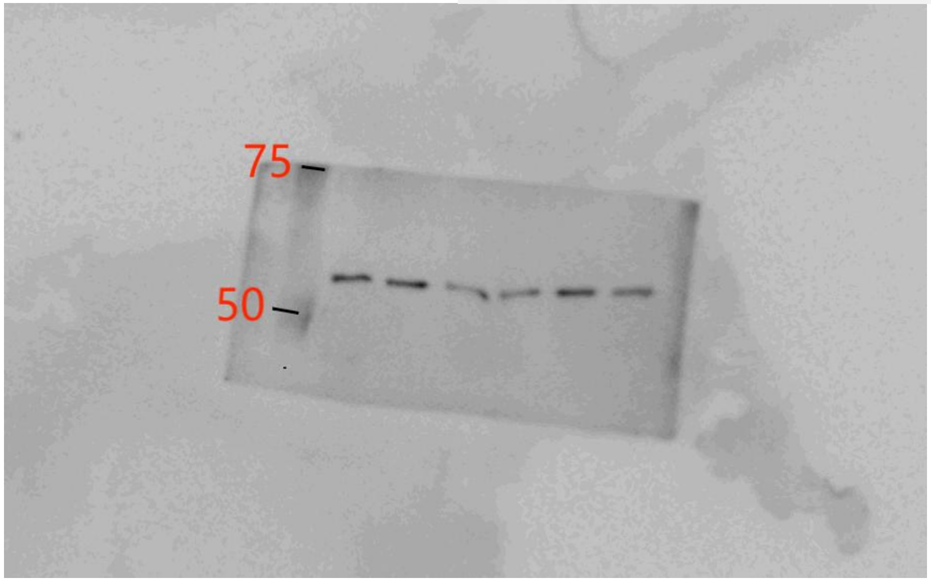

# Figure 5B α-SMA

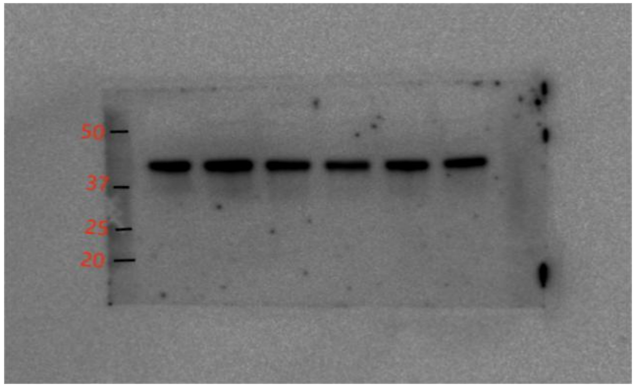


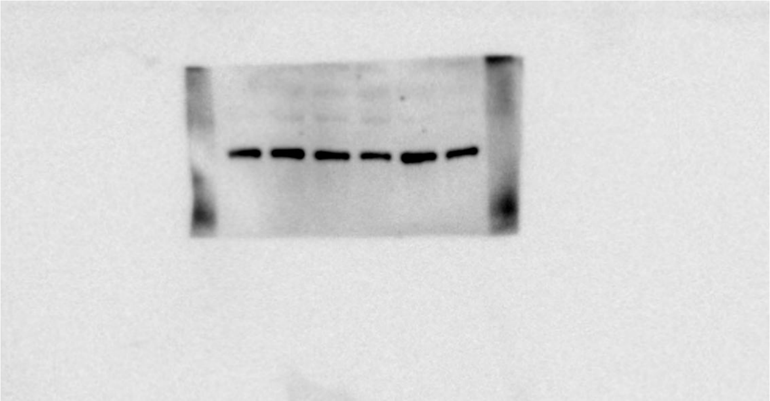

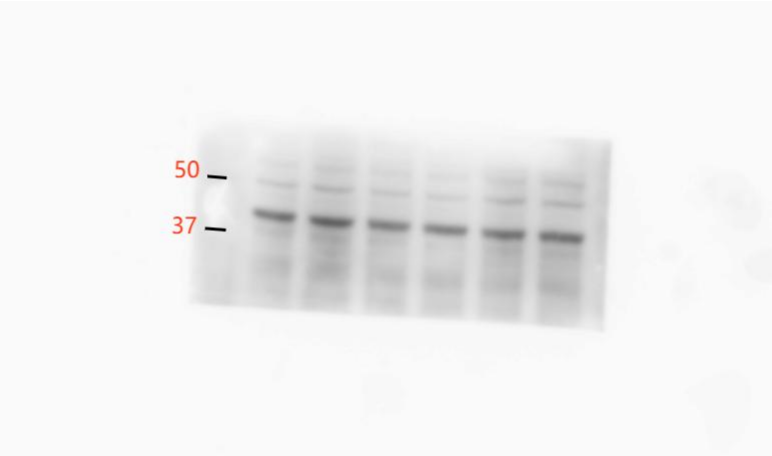

# Figure 6A β-actin

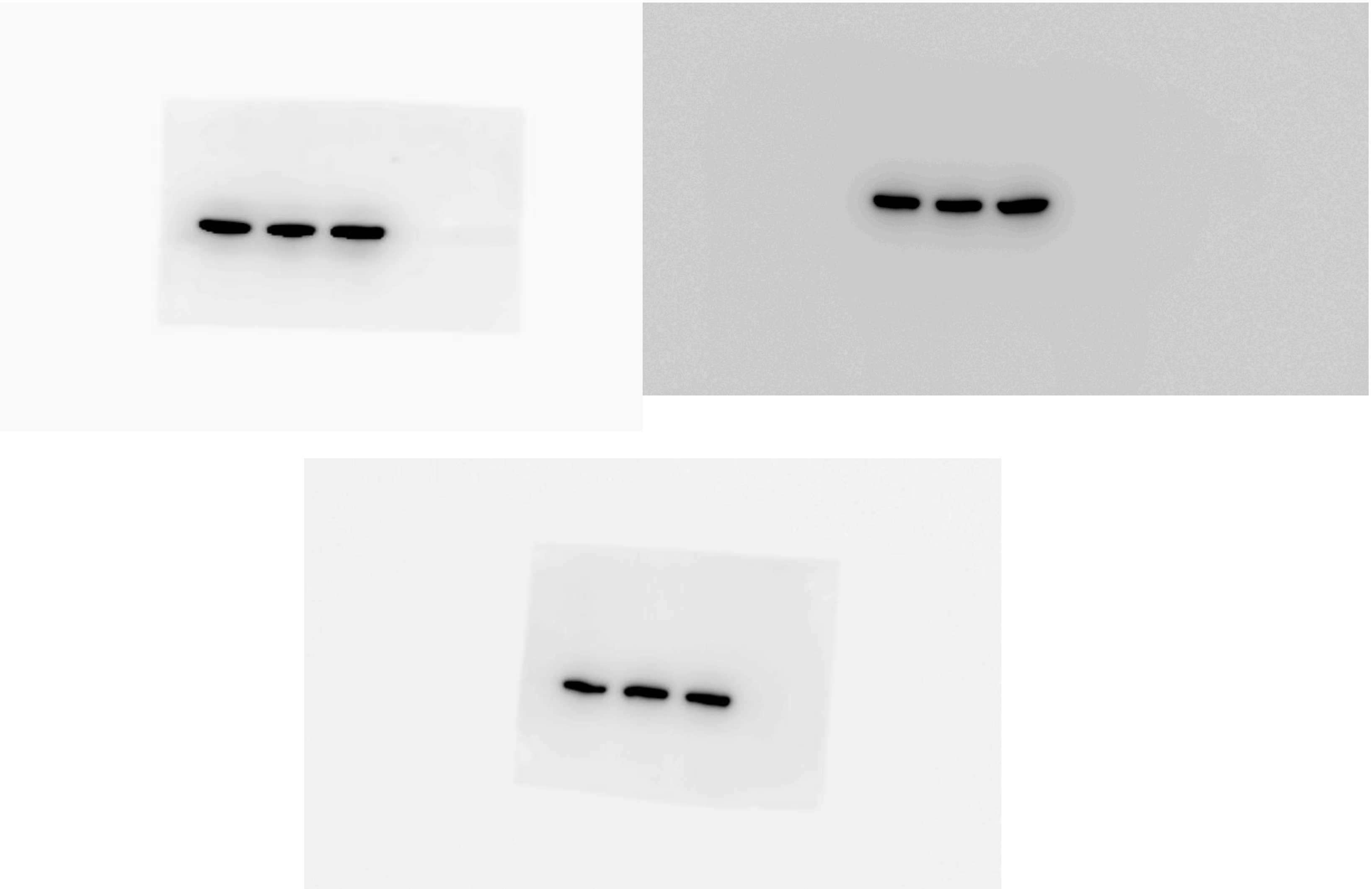

# Figure 6A  Akt

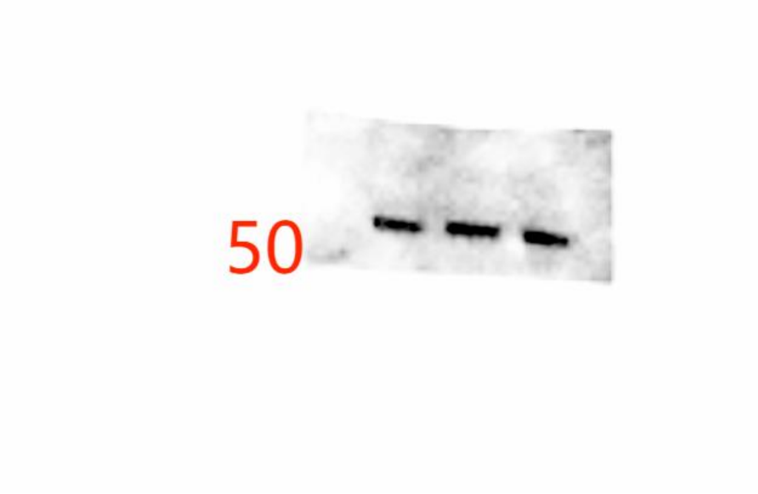


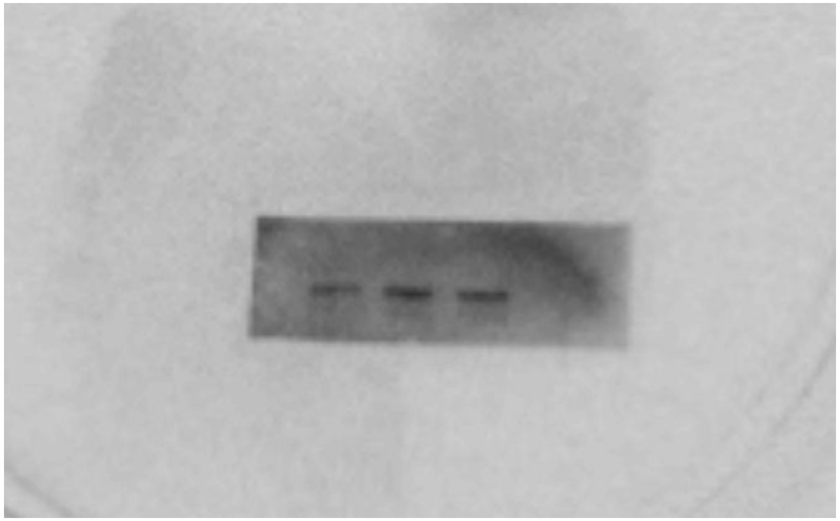

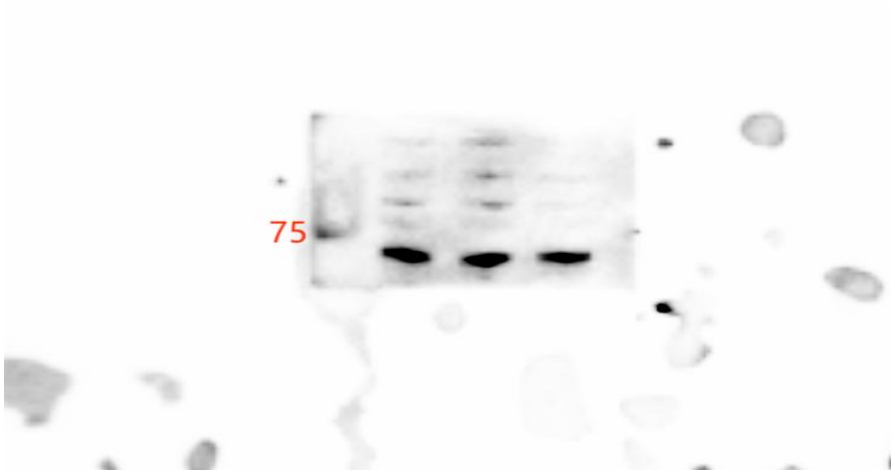

# Figure 6A p-Akt

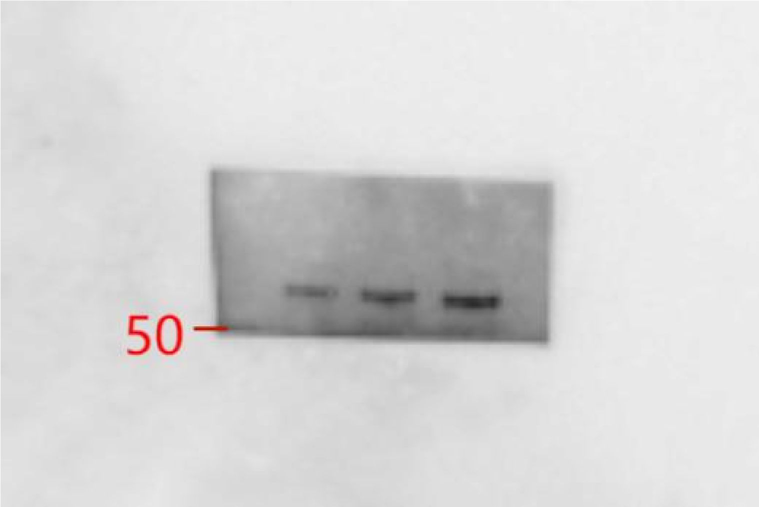


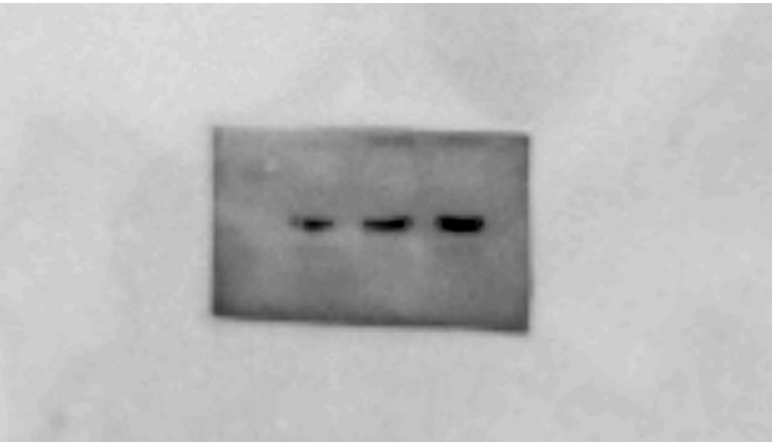

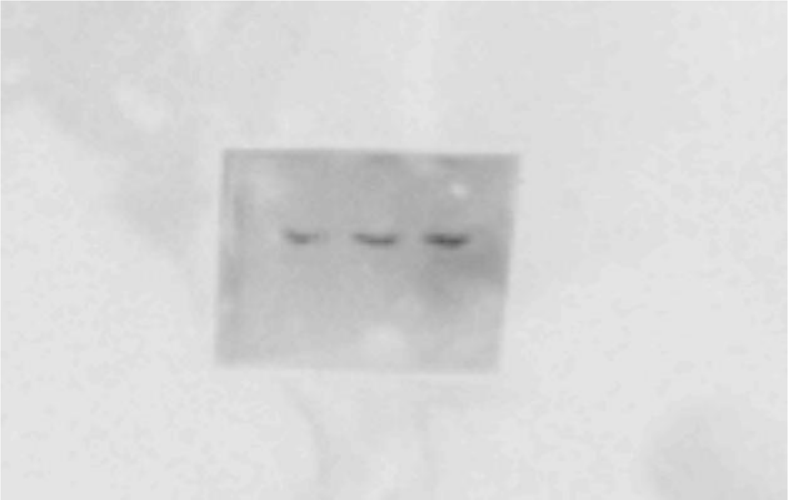

# Figure 6A ERK

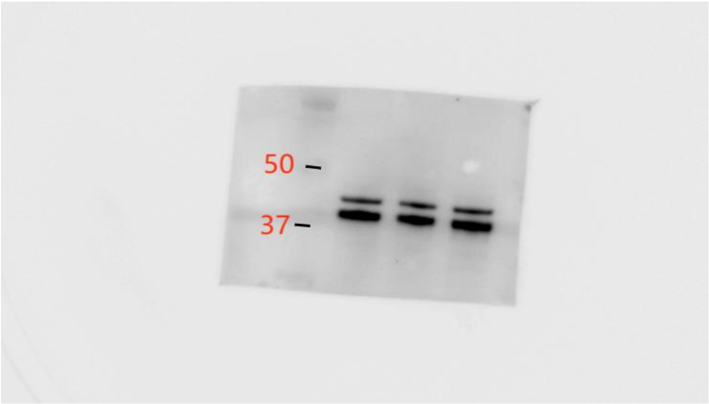


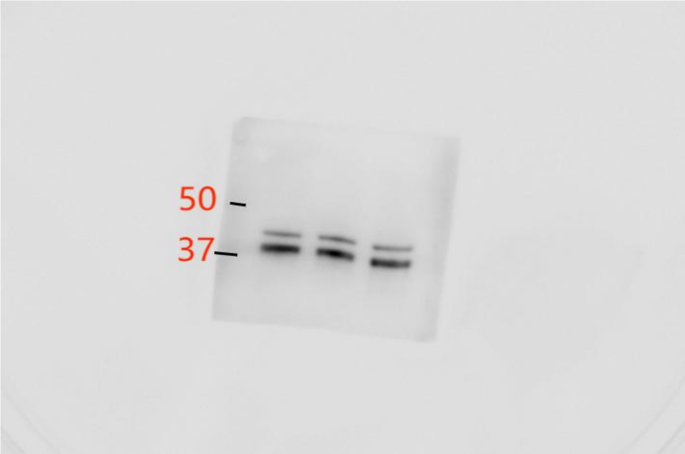


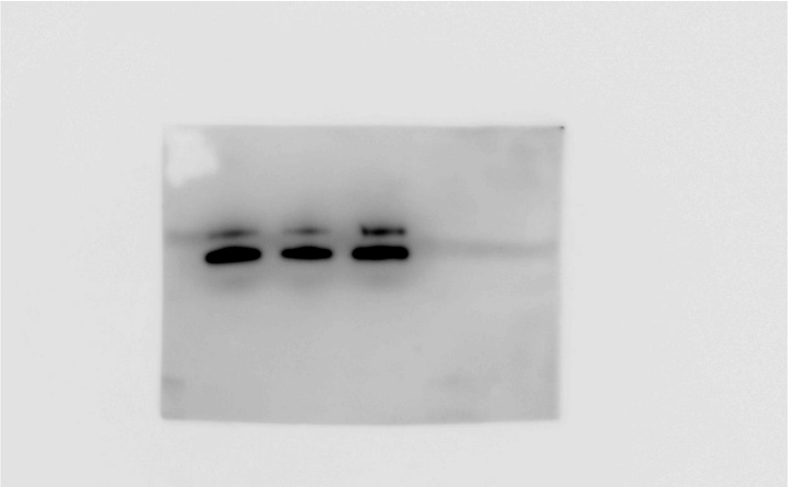

# Figure 6A p-ERK

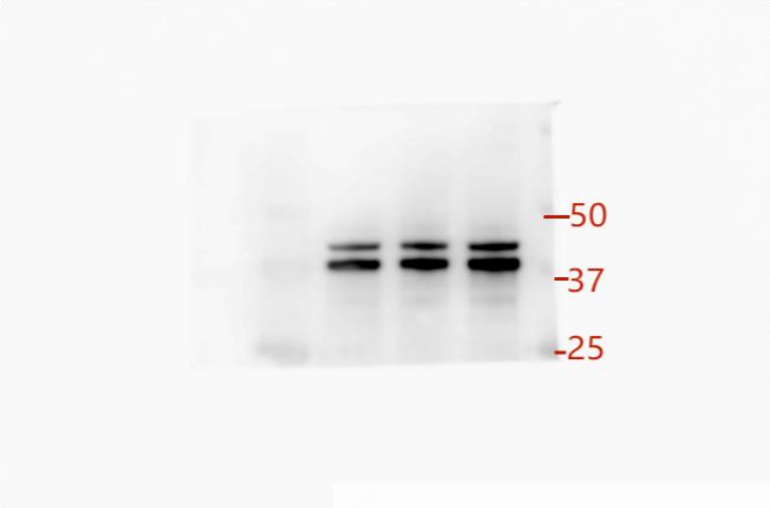


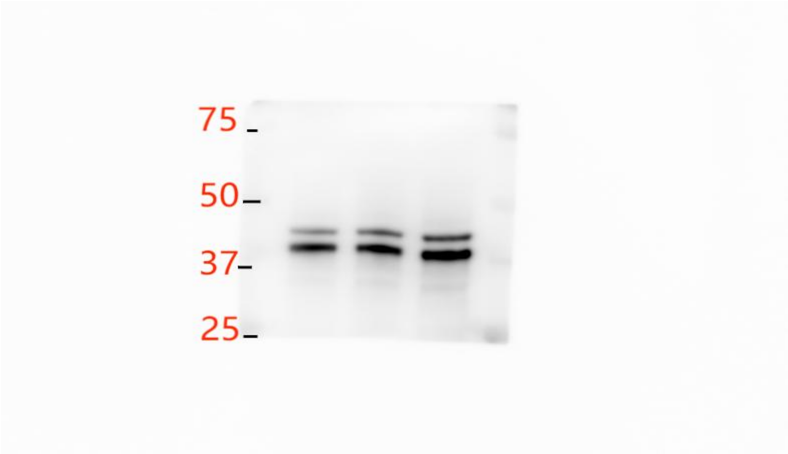


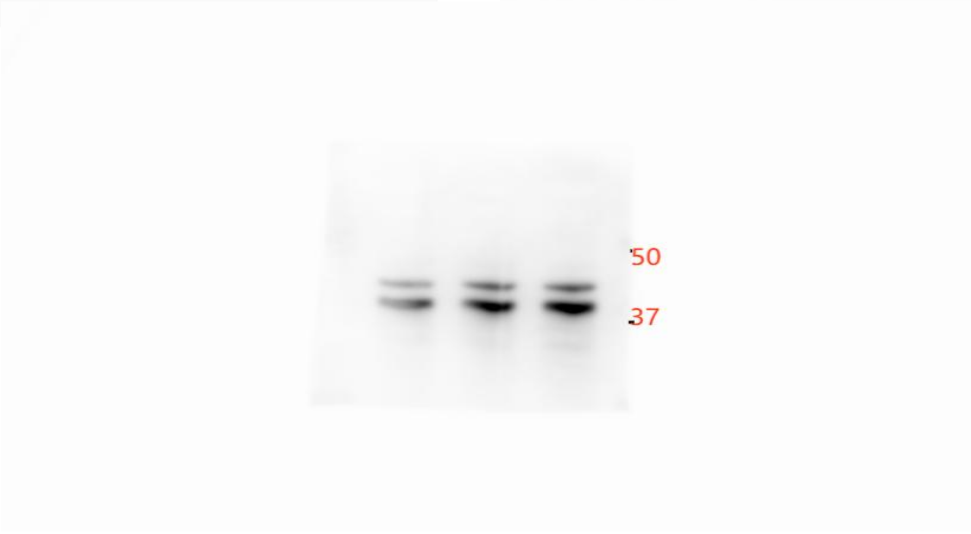

# Figure 6A p38

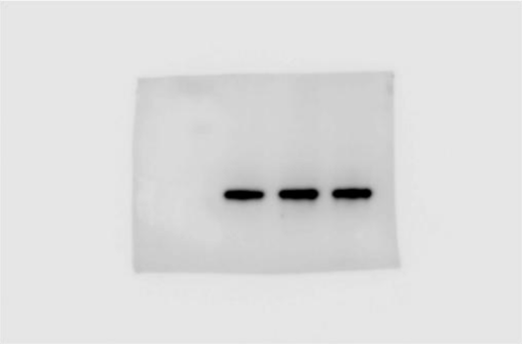

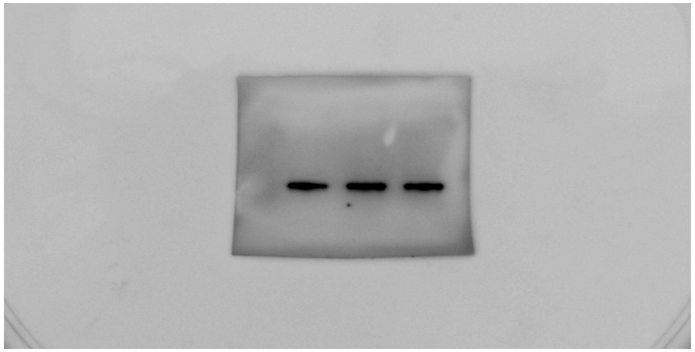

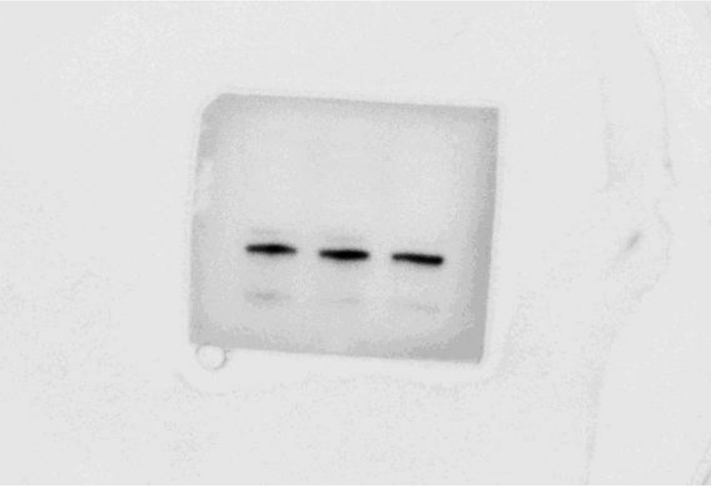

# Figure 6A p-p38

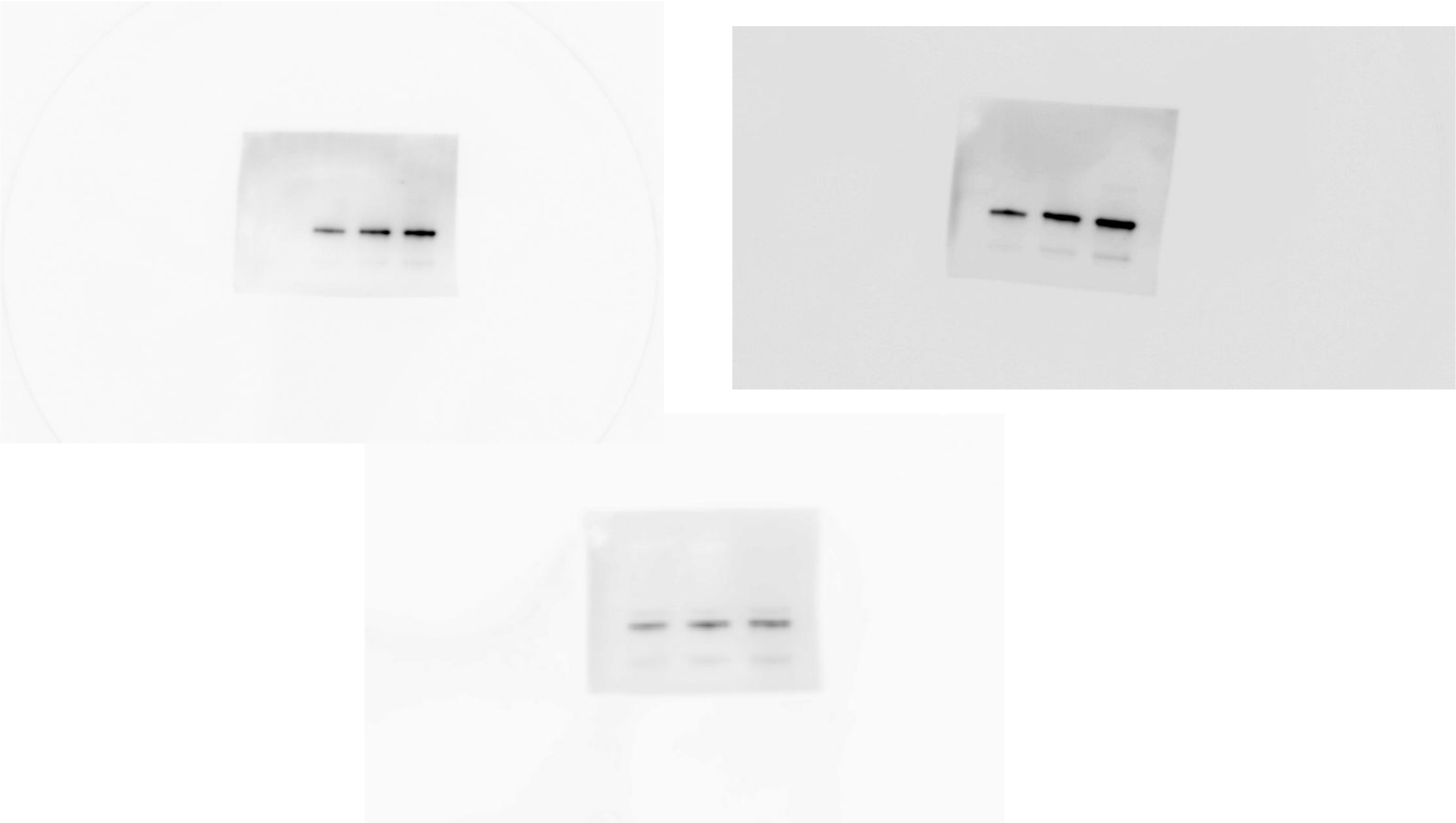

# Figure 6C β-actin

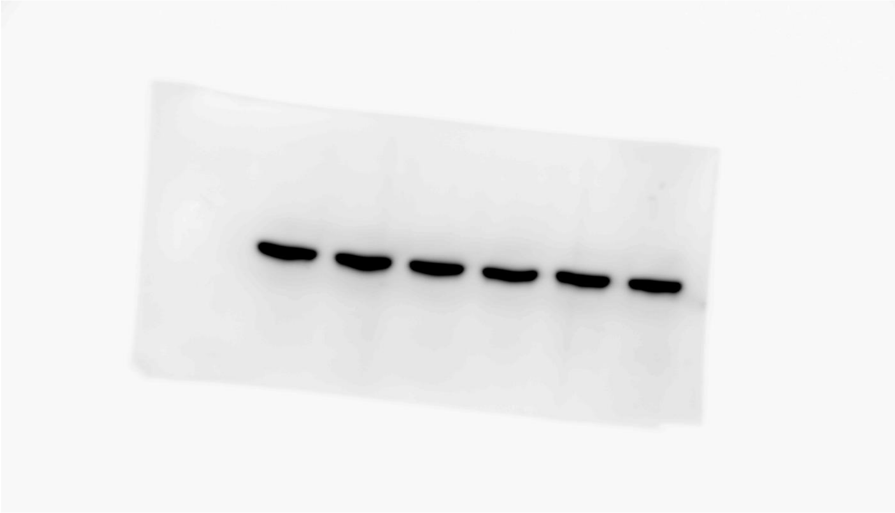

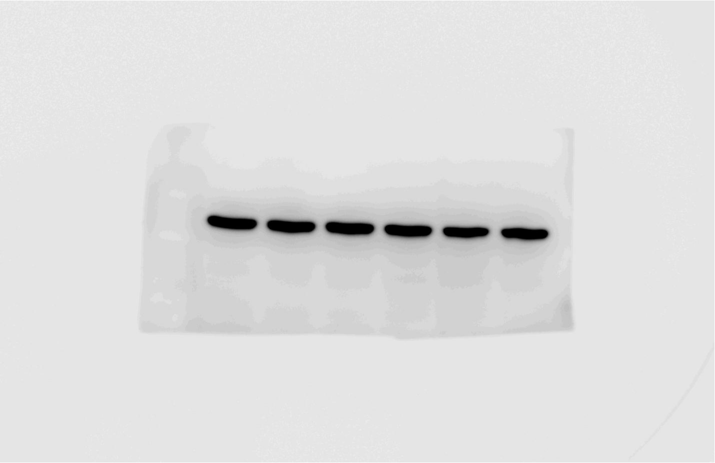

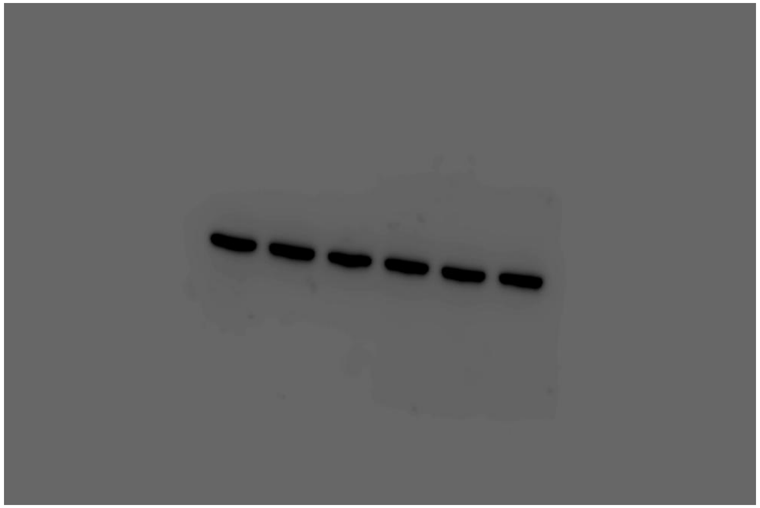

# Figure 6C Akt

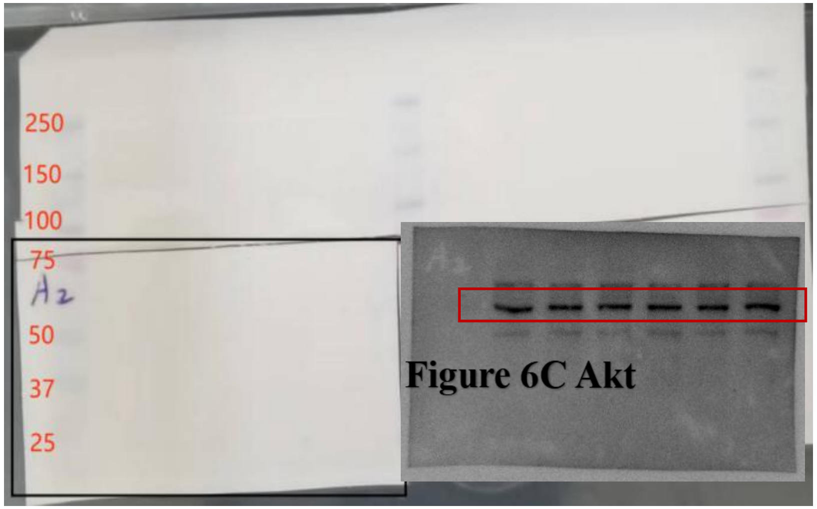


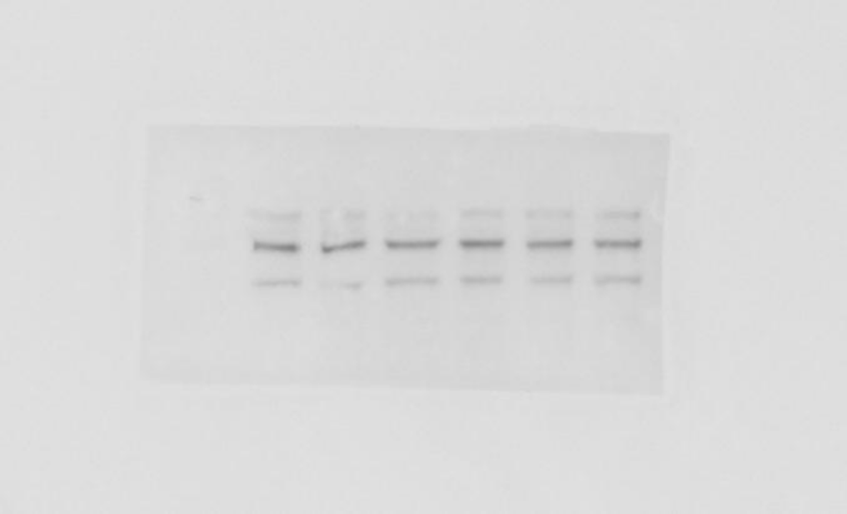

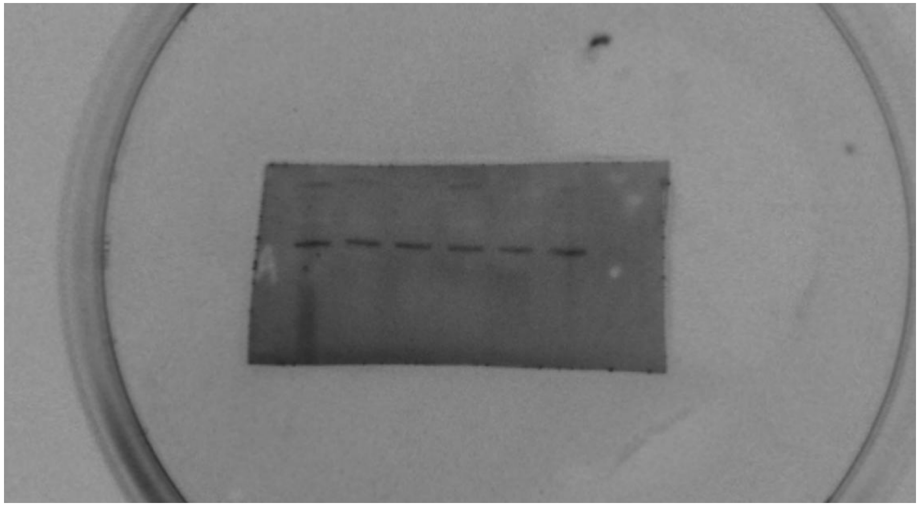

# Figure 6C p-Akt

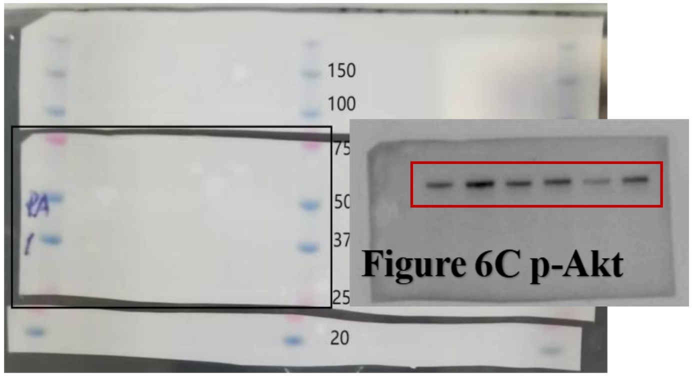


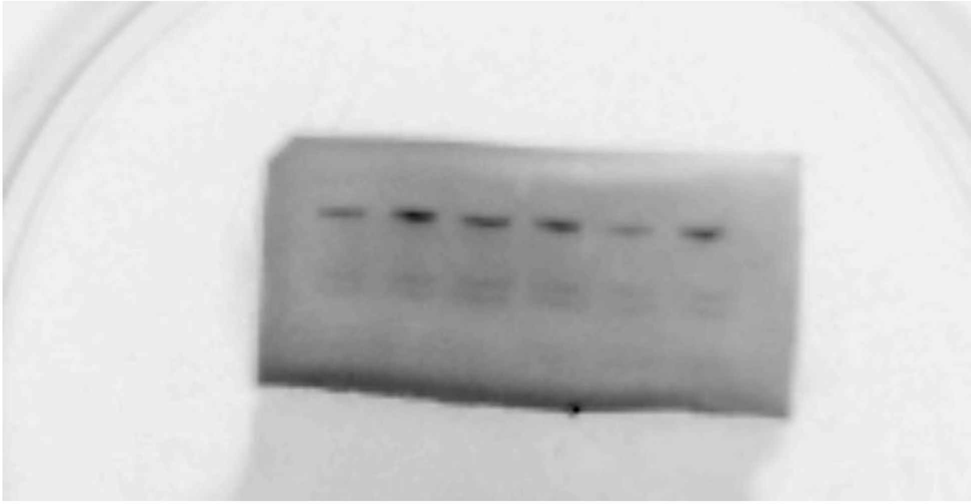

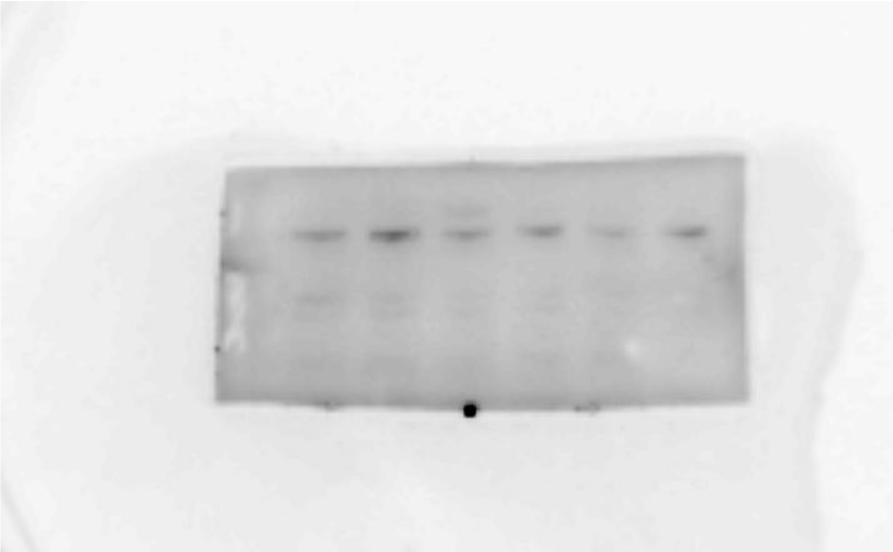

# Figure 6C ERK

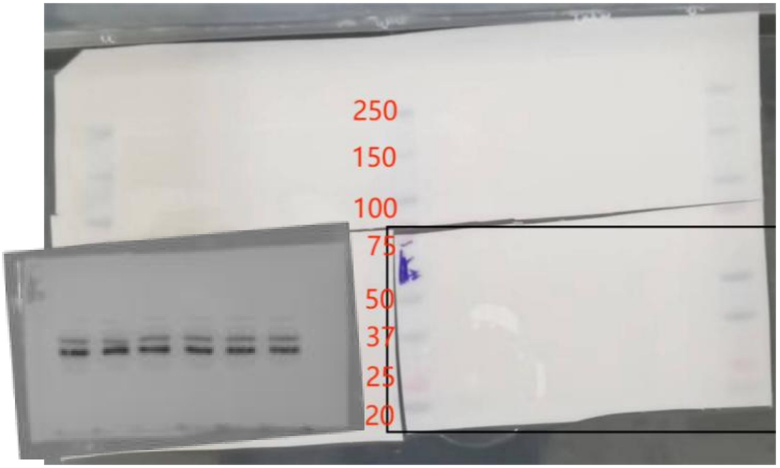


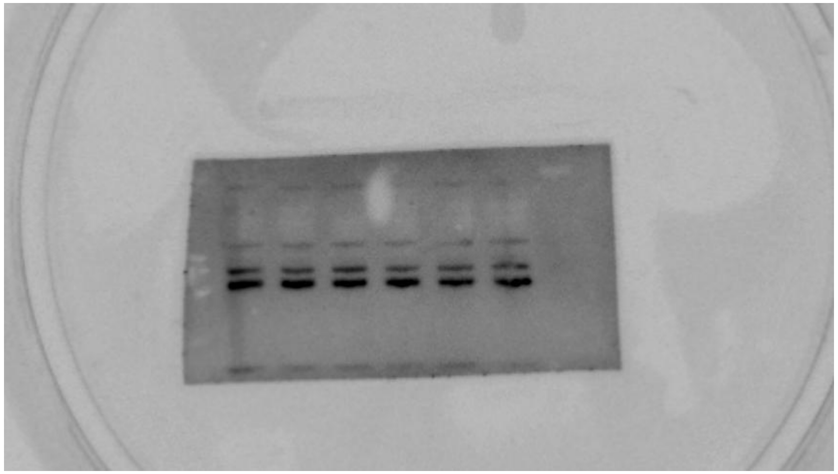

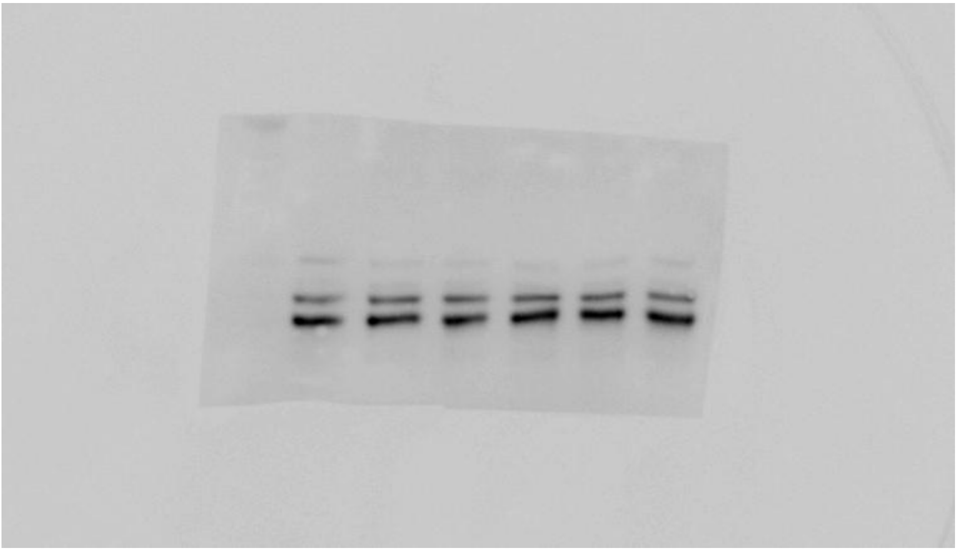

# Figure 6C p-ERK

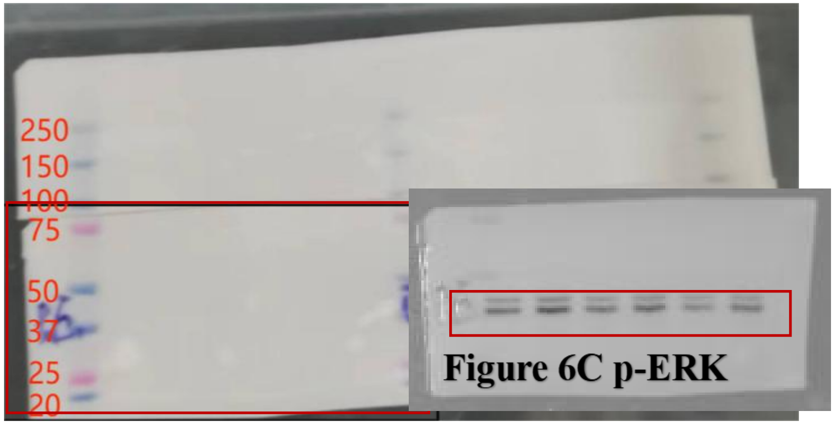


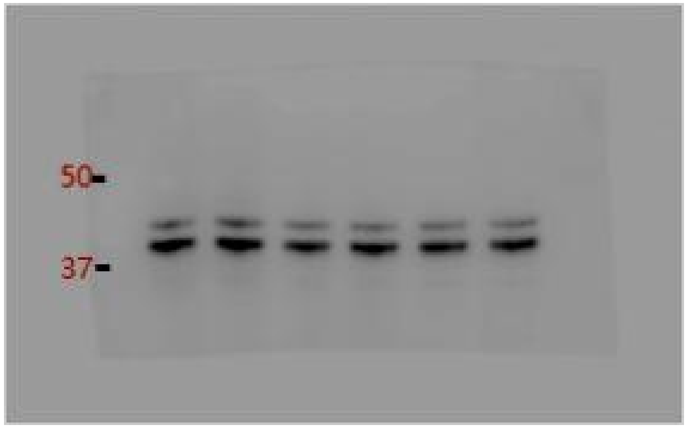


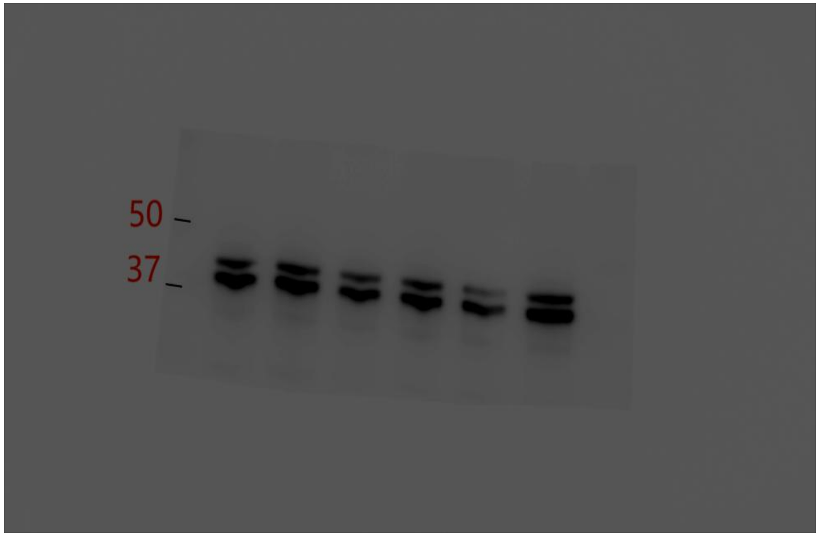

# Figure 6C p38

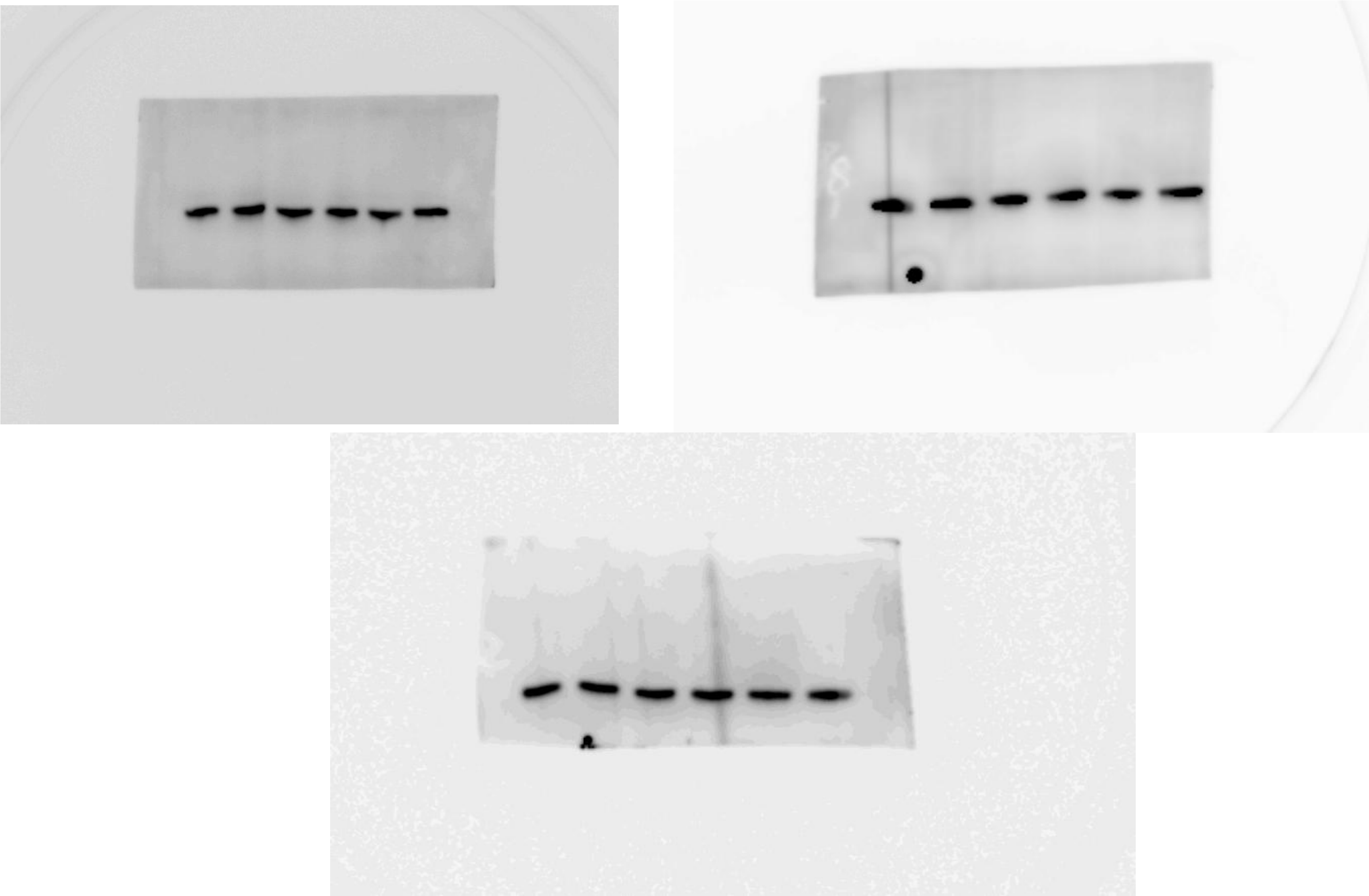

# Figure 6C p-p38

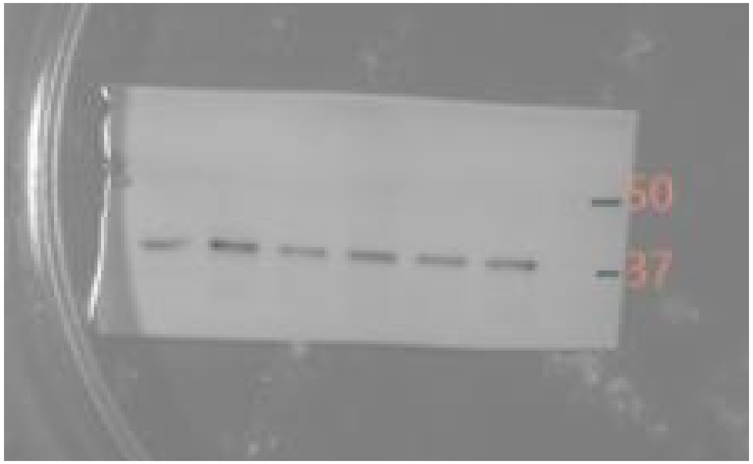


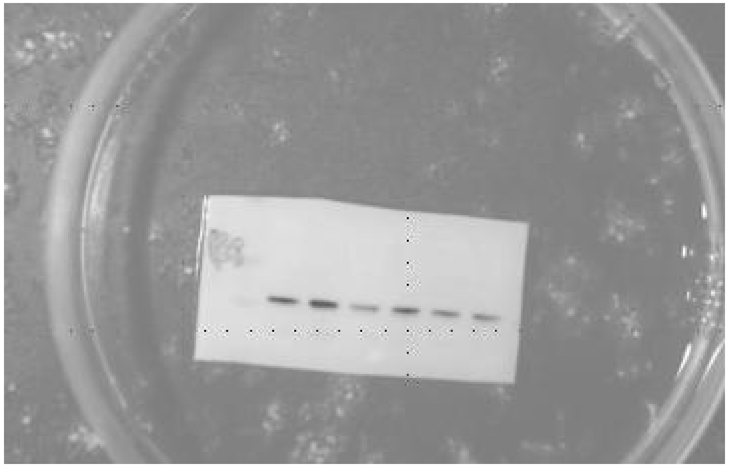

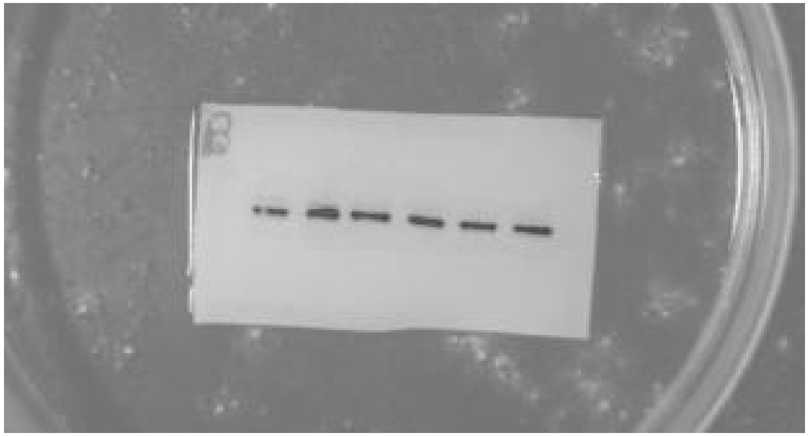

Supplement: Supplementary file 3 — Supplementary Material 3 [file 12906_2024_4483_MOESM3_ESM.pdf]
